# 60,000 years of interactions between Central and Eastern Africa documented by major African mitochondrial haplogroup L2

**DOI:** 10.1038/srep12526

**Published:** 2015-07-27

**Authors:** Marina Silva, Farida Alshamali, Paula Silva, Carla Carrilho, Flávio Mandlate, Maria Jesus Trovoada, Viktor Černý, Luísa Pereira, Pedro Soares

**Affiliations:** 1 IPATIMUP (Instituto de Patologia e Imunologia Molecular da Universidade do Porto), 4200-465 Porto, Portugal; 2 General Department of Forensic Sciences & Criminology, Dubai Police GHQ, 1493 Dubai, United Arab Emirates; 3 Faculdade de Medicina da Universidade do Porto, 4200–319 Porto, Portugal; 4 i3S (Instituto de Investigação e Inovação em Saúde, Universidade do Porto), 4200 Porto, Portugal; 5 Faculdade de Medicina, Universidade Eduardo Mondlane, 257 Maputo, Moçambique; 6 Hospital Central de Maputo, 1164 Maputo, Moçambique; 7 Departamento de Saúde Mental, Ministério da Saúde, 14 Maputo, Moçambique; 8 Instituto Gulbenkian Ciência, 2780-156 Oeiras, Portugal; 9 Centro Nacional de Endemias, CP 23, São Tomé e Príncipe; 10 Department of Anthropology and Human Genetics, Faculty of Science, Charles University, 128-43 Prague, Czech Republic; 11 CBMA (Centro de Biologia Molecular e Ambiental), Departamento de Biologia, Universidade do Minho, 4710-057 Braga, Portugal

## Abstract

Mitochondrial DNA (mtDNA) haplogroup L2 originated in Western Africa but is nowadays spread across the entire continent. L2 movements were previously postulated to be related to the Bantu expansion, but L2 expansions eastwards probably occurred much earlier. By reconstructing the phylogeny of L2 (44 new complete sequences) we provide insights on the complex net of within-African migrations in the last 60 thousand years (ka). Results show that lineages in Southern Africa cluster with Western/Central African lineages at a recent time scale, whereas, eastern lineages seem to be substantially more ancient. Three moments of expansion from a Central African source are associated to L2: (1) one migration at 70–50 ka into Eastern or Southern Africa, (2) postglacial movements (15–10 ka) into Eastern Africa; and (3) the southward Bantu Expansion in the last 5 ka. The complementary population and L0a phylogeography analyses indicate no strong evidence of mtDNA gene flow between eastern and southern populations during the later movement, suggesting low admixture between Eastern African populations and the Bantu migrants. This implies that, at least in the early stages, the Bantu expansion was mainly a demic diffusion with little incorporation of local populations.

Africa has been considered the cradle of mankind for a long time. Both genetic data (uniparental genetic markers and genome-wide diversity) and fossil evidence suggest that anatomically modern humans originated in this continent^1^, spreading later all over the globe. However, there is still a vigorous debate not only on the specific region within Africa where modern humans appeared, but also regarding the initial migrations within this continent^2^. Despite being geographically restricted to Africa before the Out-of-Africa (OOA) migration, ancestral populations most likely already displayed a strong genetic structure for at least 100 thousand years (ka)^2^,^3^,^4^, highly influenced by episodes of climate oscillation^5^.

The climate dynamics continued to contribute to African population structure after the OAA, notoriously the African Late Glacial Maximum (LGM; at ~18–16 ka^6^, later on than the northern hemisphere one at ~22–19 ka^7^), which contributed to aridity, resulting in the expansion of the Sahara desert several kilometres southwards^6^. The Pleistocene/Holocene transition (~11.5 ka) was characterized by changes in atmospheric circulation and solar radiation^6^, improving environmental conditions and leading to major human expansions in southwest Asia^8^, Europe^9^, and also in Africa (Saharan areas were recolonized^10^,^11^, allowing frequent flow across West/Central and North/South^2^,^12^,^13^,^14^). The humid conditions peaked at the Holocene climatic optimum (~9–6 ka), when Sahara desert virtually disappeared and the Chad lake was seven times larger than today^14^. A shift to aridity occurred later in the Sahara, at ~6 ka^15^.

More recently, the African genetic and cultural landscape was deeply affected by an event known as the Bantu expansion. The expansion of Bantu-speakers is thought to have started in the Grassfields region between southeast Nigeria and western Cameroon and taken two main routes from its starting point: a western route, throughout the west coast of Africa, having arrived to Angola, South Africa and Botswana around 3.5 ka, and an eastern route, towards the Great Lakes in Eastern Africa, reaching the region of Uganda about 2.5 ka, where they remained for a couple thousand years, expanding later into the south, reaching Mozambique by ~1.8 ka^16^,^17^,^18^. The Eastern route is of particular interest to study potential crossings between migrants and local eastern populations (namely Nilotic and Cushitic people), during the period in which the Bantu people were stationed in the Great Lakes region. Linguistic differences between eastern and western Bantu languages seem to mirror the two routes of expansion, but, recent evidence suggests a later split of Eastern and Western Bantu^19^. Either way, the Bantu expansion probably forced the retreat of contemporary local sub-Saharan populations: the San were further confined to the South towards the Kalahari desert and kept their typical Khoisan languages (with click consonants) and ethnic identity, and the Pygmies, on the other hand, were pushed deeper into the forests and eventually some adopted Bantu languages^20^.

Recent methodological and technical advances led to the emergence of genome-wide (GW) studies, whose main advantage for demographic inference is allowing us to identify and quantify admixture between populations of distinct ancestries^21^. However, current GW dating methods are still limited in dissecting between several migration waves, usually leading to the identification of a single event of average/young age (discussed in^22^). Mitochondrial DNA (mtDNA), on the other hand, is only maternally inherited, but due to its fast mutation rate, accumulates variation fast enough amongst different locations, to make it a suitable molecular marker for the phylogeographic approach. Since reliable mtDNA mutation rates have been calculated, it is possible to frame the various demographic events within distinct time periods^23^. A lineage-based approach can thus provide insights into the demography of populations and reveal patterns that would otherwise be dismissed, and has proved particularly useful to resolve the old debate regarding the Bantu expansion: the identification of specific lineages suggested that the expansion of Bantu languages was due to the migration of Bantu-speakers, rather than just a cultural diffusion^19^, as previously thought.

Previous studies based on hypervariable segment I (HVS-I) diversity have shown that haplogroup L2 played a major role in the Bantu migration^17^,^18^,^24^. MtDNA haplogroup L2 is the sister branch of the Eastern African L3′4′6 clade that contains all the OOA diversity within haplogroup L3. While L3′4′6 originated in Eastern Africa^22^, haplogroup L2 probably originated in Western Africa but is nowadays widespread across the continent; it is highly frequent in many regions, such as in Western/Central and Southeast Africa (probably associated with the Bantu expansion that occurred in the last few millennia) and in Northwest, most likely due to trans-Saharan slave trade^18^,^25^. Together with haplogroup L3, it represents ~70% of sub-Saharan mtDNA variation but despite its high frequency and wide distribution, L2 was not involved in the OOA^26^, since most likely it was not yet arrived in Eastern Africa by that time.

The demographic history of L2 is not yet completely understood, especially concerning the age of the expansion into Eastern Africa, a region that might have acted as a refuge during some severe episodes of climate oscillations over the last hundred thousand years^27^. One possibility is that the expansion of L2 to the East, most likely as with the expansion to the South, was related with movements of Bantu-speaking populations. However, in the regions of highest frequency of L2 in Eastern Africa (over 30%, in the area of Sudan and Ethiopia)^13^ there are no records of Bantu groups. Furthermore, recent evidence from HVS-I^13^ suggests that this haplogroup might have first expanded to Eastern Africa much earlier, possibly due to the improvement of climate conditions during the early Holocene. This signal was also observed with Bayesian analysis of L2 (and L2a) complete sequences^28^. Moreover, particular clades of L2a and L2c suggest an expansion, possibly along the Sahel corridor, after the LGM^18^. Migrations at this time frame are also observed in branches of other African haplogroups, such as L0a, L1b and L3f^2^,^12^,^18^,^29^.

Despite being spread across different regions, most of the haplogroup L2 sequences available in online databases are either from Western or Southern Africans or from African-Americans. We aim to better understand the phylogeographic patterns of L2 by improving its phylogeny based on complete sequence information especially for Eastern Africa, a region poorly characterized for L2 clades. This increased resolution will enable us to ascertain about the intensity of the gene flow from Eastern populations to the Bantu migrants towards south. The L2 complete sequence analysis was complemented by a similar analysis for haplogroup L0a (also present in Central and Eastern Africa by the time of the Bantu expansion^2^) and a HVS-I population-based approach.

## Results

### Phylogeography of haplogroup L2

**Figure 1:**
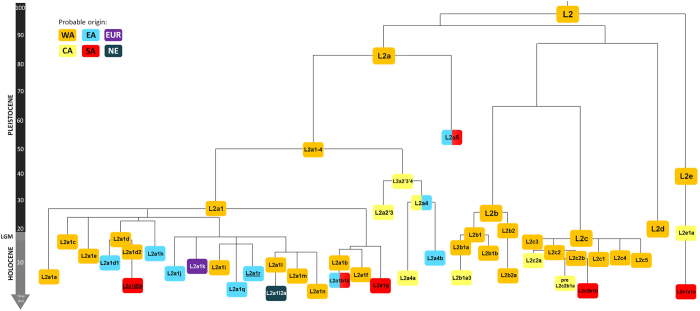
Schematic phylogeny of mtDNA haplogroup L2, based on ML age estimates. Colour scheme corresponding to the probable origin of each clade (WA – Western Africa, CA – Central Africa, EA – Eastern Africa, SA – Southern Africa, EUR – Europe, NE – Near East/Arabian Peninsula), new branch labels proposed in the present study are underlined.

A schematic tree of haplogroup L2 is shown in Fig. 1. The complete phylogeny is shown in Supplementary Table 1, including *ρ* age estimates (considering both the complete mitochondrial genome and the synonymous clocks), ML age estimates and Bayesian age estimates based on a relaxed molecular clock for the main nodes. We tested the molecular clock with a likelihood ratio test^30^, which confirmed previous evidence of clock violation for this haplogroup^26^,^31^,^32^. In this sense, the Bayesian molecular clock, which allows rate variation, is more accurate. However, ML age estimates are also restrained by the tree structure and the overall mtDNA clock, that generally does not show strong violations^33^. ML age estimates are comparable to the Bayesian estimates. This is seen by the correlation between the estimated branch lengths between both analyses (ratio close to 1) (Supplementary Fig. 1a). Nevertheless, when comparing the age estimates themselves, for younger nodes the Bayesian analysis provides substantially higher age estimates (Supplementary Fig. 1b). Considering that the clock used for Bayesian inference was based on the age of haplogroup L3 (~70 ka)^12^, probably inappropriate for the period of interest (mainly Holocene), and the similar estimated branch lengths in the Bayesian inference and ML, we consider that the time dependent clock employed in the ML analysis containing the correction for purifying selection developed by Soares *et al.*^23^ is more appropriate for such short time scales, particularly for ages below 20–15 ka (Supplementary Fig. 1b), as it showed previously a good correspondence for the recent colonization of the Pacific^34^. All age estimates for the L2 clades mentioned in this chapter are shown in Table 1.

**Figure 2:**
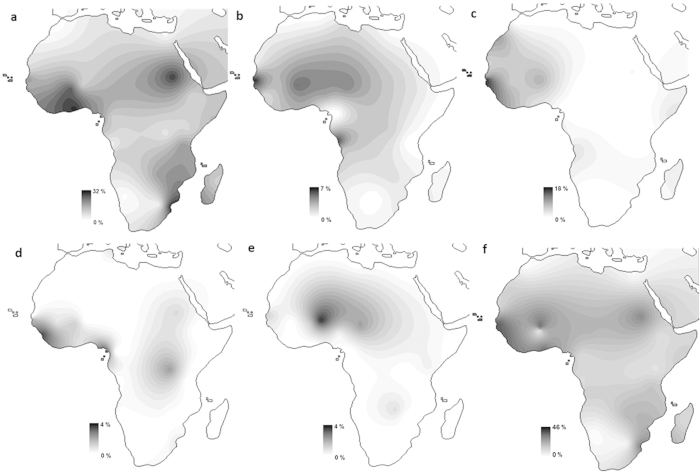
Frequency distribution maps for mtDNA haplogroup L2. Maps for L2a (**a**), L2b (**b**), L2* (**c**), L2d (**d**), L2e (**e**) and total L2 (**f**). The map was obtained from the website www.outline-world-map.com.

In general, L2 has a very complex structure. L2 divides into five main branches (L2a–e) (Fig. 1). The earliest split of L2 (99.1 ka in ML; 78.6 ka in the Bayesian inference (BI)) is between L2e and L2a–d (L2a’b’c’d). Regarding L2e (38.9 ka in ML and 34.3 ka in BI (Table 1)), both the frequency distributions (Fig. 2e) and its most basal branches (Supplementary Table 1) suggest a Western African origin. A southern sub-branch, labelled in this study as L2e1a1a, dating to about 2 ka, was probably involved in the Bantu migration.

L2a splits from L2b-d (L2b’c’d) at ~93.5 ka in ML and ~73.7 ka in BI. L2a (~84.4 ka in ML; ~66.1 ka in BI), is geographically widespread and highly frequent throughout Africa (Fig. 2a) and accounts for more than 70% of all L2 branches^18^,^26^, with peaks of frequency in Ghana, Sudan and Mozambique. L2a divides into five branches (L2a1-5), with the earliest split between L2a5 and L2a1-4. L2a5 is practically restricted to Southern Africa but also detected at lower frequencies in Eastern Africa. It dates to ~56.2 ka in ML and ~46 ka in BI and suggests a migration into Eastern or Southeast Africa between ~95 ka and 45 ka (considering the range of age estimates for L2a and L2a5). Since it is found only in Bantu speakers, this clade was probably assimilated by Bantu migrants. L2a1-4 splits between L2a1 and L2a2-4. L2a2'3'4 (~36.5 ka in ML; ~32.3 ka in BI) seems to have a more Central African distribution, with a clear association to Pygmy groups. The major split with other regions is within L2a4 (27.6 ka in ML and 23.6 ka in BI), separating a Pygmy specific branch (L2a4a) and an Eastern African branch, L2a4b (12.5 ka in ML and 10.7 ka in BI), suggesting a Late Pleistocene/Early Holocene entrance in Eastern Africa.

L2a1 (26.5 ka in ML and 29.6 ka in BI) is the most complex sub-clade within L2a and it harbours lineages from all African regions, as well as lineages from other continents, including non-African branches, such as L2a1l2a (connected to Ashkenazi Jewish Diaspora^35^,^36^), and the exclusively European L2a1k^37^. Phylogenetic reconstruction of L2a1 is often difficult due to high levels of homoplasy. Major splits within L2a1 defined by homoplasic positions (143, 16189, 16192 and 16309) exist for parsimonious reconstruction purposes but will not be considered in the text. L2a1a has clearly a Western/Central African origin and distribution, with many sub-clades suggesting a recent Bantu migration southwards, and is hardly present in Eastern Africa. This pattern is also visible in L2a1c, L2a1f and L2a1i. L2a1e and the minor clade L2a1m exist essentially only in Western/Central Africa. L2a1l displays a similar pattern in sub-Saharan Africa, but with the peculiarity of a sub-branch present in Ashkenazi Jews, L2a1l2a^35^. L2a1b again shows an origin in Central Africa, but subclade L2a1b1a dating to 6.9 ka in ML is present in Southern Africa and has a few lineages in Eastern Africa (mainly Somalia). It might have moved earlier to the East in the Early Holocene and incorporated later by Bantu migrants. L2a1d splits into an Eastern African sub-clade (L2a1d1) at ~10.6 ka and L2a1d2 that shows a split between a Western African lineage and a Southern African clade dating to about 7 ka that contains the star-like L2a1d2a clade dating to 3.7 ka. Other clades show additional evidence of an early migration into Eastern Africa, like L2a1h and L2a1j. We detected a new clade specific to Somalia, L2a1r, at 7.3 ka. The clade L2a1 + 143 shows several basal Eastern African lineages (together with Near Eastern and Arabian lineages) that indicates a migration in the Early Holocene. Minor clades, namely L2a1g and L2a1q, are present in Bantu-speaking populations in the South and, although they were not detected in Western/Central Africa, their lower age suggest a direct involvement in the Bantu expansion.

The major split within L2b’c’d is between L2b’c and L2d (~86.7 ka in ML; 65.1 ka in BI). L2d (~19.0 ka in ML ~16.2 ka in BI) is a rare clade dominated by basal western branches, supporting the overall origin of L2 in Western Africa. Lastly, L2b and L2c split at 65.3 ka in ML and 49.7 ka in BI. L2b (~26.0 ka in ML and ~24.5 ka in BI) displays four branches, all with probable origin in Western/Central Africa. Two sub-branches, L2b1a3 and L2b2a, could have been involved in the Bantu expansion. L2c (~17.2 ka in ML; ~18.3 ka in BI) is essentially western as reported elsewhere^38^,^39^, reaching the highest frequencies in Gambia, Sierra Leone and Cape Verde (Fig. 2c). Two branches, L2c2a1 (5.9 ka in ML) and L2c2b1b (~2.5 ka in ML) are associated to southern populations and most likely related to the Bantu expansion.

In order to assess if the movements observed in the L2 tree were accompanied by the increase in the effective population size (Ne) associated to the haplogroup, we computed Bayesian Skyline Plots (BSPs). The BSP for total sub-Saharan African L2 dataset shows two moments of increment in the N_e_ associated to L2 (Supplementary Fig. 2a): ~11.5 ka, corresponding to the Pleistocene/Holocene transition and ~5 ka, probably associated to the Bantu expansion.

### Early Holocene gene flow between Central and Eastern Africa

In a previous study based on HVS-I, a probable major migration of L2 lineages in the Pleistocene/Holocene transition was discerned^13^. We reproduced the network analysis for the present HVS-I dataset and the three main nodes detectable at HVS-I level (L2a root, L2a + 16189, L2a + 16189 + 16192) showed founder ages^40^ between 7 ka and 15 ka, supporting a movement into Eastern Africa substantially before the Bantu expansion. L0a also showed a similar pattern but in the opposite direction^2^, suggesting that bidirectional gene flow occurred between Central and Eastern Africa in the Pleistocene/Holocene transition period. Since phylogeographic inferences based on HVS-I alone can be misleading we further tested the patterns with whole-mtDNA genomes.

From the 801 complete L2 sequences contemplated in our analysis, only 39 are from EA. Considering that 24 were sequenced in this study, it is evident how poorly this region was represented in past studies. L2 complete sequences, similarly to HVS-I, indicate that most lineages arrived to Eastern Africa in the Early Holocene or Late Pleistocene. L2a1b contains Somali lineages, whose founder age in Eastern Africa is 7.9 ka [1.5; 14.5] and the Eastern African L2a1d1 dates to 10.6 ka. L2a1h, probably with Eastern African origin, dates to 14.4 ka while L2a1r, a newly labelled Somali clade, dates to ~7.3 ka. Additionally, around 20% of Eastern African lineages cluster within the L2a1 + 143 branch (24.8 ka in ML). A founder age of this cluster suggests a migration time at 14.8 ka [10.2; 19.5], pointing to a migration in the Late Glacial or postglacial period. Overall, as predicted by HVSI-I data, most of the L2 lineages entered Eastern Africa between 15 and 7 ka.

A BSP restricted to Eastern Africa shows a N_e_ increase in the Pleistocene/Holocene transition (Supplementary Fig. 2c), despite the low number of eastern samples. A signal of expansion was also visible at ~10 ka in the BSP containing Western/Central African lineages (Supplementary Fig. 2b) which could be expected considering that the migration of L2 lineages into Eastern Africa might have been triggered by an expansion of these lineages in the source.

### Bantu expansion

**Figure 3:**
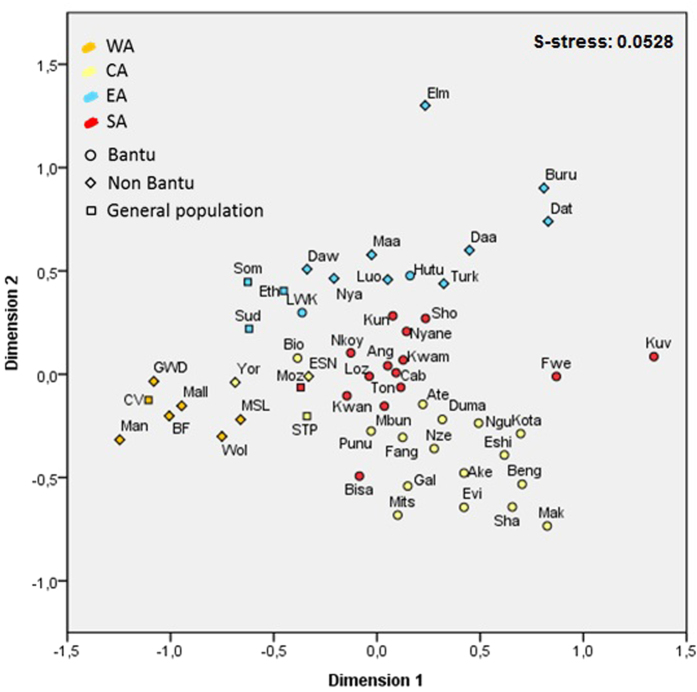
MDS plot based on Slatkin’s linearized F_ST_. Colour code: WA – Western Africa, CA – Central Africa, EA – Eastern Africa, SA – Southern Africa.

Most of the typically Eastern African sequences (many L3, L4, L5 and L6 clades) are not frequent in Southern Africa, whereas L0a and L2 are also very common in Southern Bantu-speakers (Fig. 3). Considering their wide distribution across Central and Eastern Africa, probably established in the postglacial period, L2 and L0a southern clades could have different origins: trace their immediate origin to Central Africa in the last 5 ka, indicating they were carried by Bantu agriculturalists; or coalescence with Eastern African branches, suggesting direct gene flow from non-Bantu populations to the migrating Bantu-speakers in Eastern Africa (possibly in the Great Lakes area).

Taking advantage of the recently well characterized Southeast African whole mtDNA pool^41^,^42^,^43^, we identified that most (88%) L2 Southeast African branches perfectly mirror the Bantu expansion, deriving from Central African clades in the last few millennia and mostly displaying a star-like pattern, a signal of a recent expansion. Examples of such clades are found in L2e1a1a (1.8 ka), L2c2b1b (2.5 ka) and L2a1a2a1a (5.3 ka). These clades do not display any Eastern African representatives (Supplementary Table 1), or in the few exceptions the eastern samples result of gene flow from settled eastern Bantu populations, rather than the other way around.

There are, nevertheless, episodic minor southern clades presenting an Eastern origin (e.g. the minor L2a1 h). In L2a1d2 there is a Southern African branch deriving from Western Africa and containing the major sub-branch L2a1d2a (labelled in this study, ~3.7 ka), with very strong star-like pattern. However, one Zambian sample places L2a1d2a ancestry in Southern Africa at 7 ka. Considering the overall pattern, either a closer Central African representative is missing by chance, or the possibility of sequencing errors in the Zambian ancestral sample cannot be excluded.

But the most singular case representing the bulk of southern L2 lineages not displaying a Central African Bantu origin is L2a5 (56.2 ka in ML; 46 ka in BI). L2a5 is present essentially in Southeast Africa, despite having been also detected in Eastern Africa in the HVS-I dataset. Its split with other L2a lineages dictates the root of L2a and suggests a movement from Central Africa to Eastern or Southeast Africa between the age of L2a (84.4 ka in ML, 66.1 ka in BI) and the age of L2a5. Movements from Eastern to Central Africa, probably associated to climate change^44^, might have carried ancestral lineages of L3e and L3b’d around 50-40 ka^12^ and it is not unlikely that movements occurred in both directions. This clade being incorporated by Bantu-speakers represents the major detected input of autochthonous lineages (either in Eastern or in Southeast Africa), apart from L0d and L0k further south. One possibility is that this lineage was already present in the early Bantu populations moving South after the standing point in the Great Lakes in Uganda. Since we have no whole mtDNA information on Uganda we cannot exclude it as the possible origin of L2a5.

The BSP for Southern Africa (Supplementary Fig. 2d) shows a rapid increment in L2 N_e_ ~2.5 ka, consistent with the results of the overall L2 data and with a rapid increase during the Bantu expansion. Again, the signal was also detectable in the BSP for Western/Central Africa (Supplementary Fig. 2b), which presents a second peak at ~5 ka (Supplementary Fig. 3). The Southern African dataset is the only one with a small increment between 60 and 70 ka (Supplementary Fig. 3), which could be due to the before-mentioned L2a5 in the region.

Like L2, the distribution of L0a was for a long time linked to Bantu movements, but recent evidence supports an earlier expansion of L0a to Central Africa during the Pleistocene/Holocene transition^2^,^28^ and a later incorporation in the Bantu expansion southwards. An updated L0a phylogeny is shown in Supplementary Table 2. Time to the most recent common ancestor (TMRCA) estimates for the branches mentioned in the text are also shown in Table 1. L0a shows great parallelism with L2, being the main difference the principal direction of the movement in the postglacial period (L0a originated in Eastern Africa). Nearly 75% of the Southern African L0a lineages show a Central African origin (Supplementary Table 2), despite the eastern origin of this haplogroup. Clades like L0a1a2 and L0a1b1a have a Central African origin and an association with the Bantu expansion, without the involvement of Eastern African lineages. However, one frequent branch, L0a2a2a, shows evidence of an Eastern African origin and probably represents the assimilation of Eastern lineages by the migrating Bantu groups.

### Population analysis

In order to confirm the dynamics of Bantu and Eastern populations observed within haplogroup L2, we performed a HVS-I population-based analysis. The MDS plots (Fig. 3 and S4) display Young’s S-stress values that guarantee that they are accurately portraying relationships between the populations^45^. The first dimension (Fig. 3) does not consistently differentiate any comprehensive group based on geography or language. Nevertheless, it separates Western from Central Africa, with Southern Africans grouping mainly with Central Africans. The most divergent groups, which keep their differentiated positions in the MDS excluding L2a and L0a (Supplementary Fig. 4), are the Kuvale, Bantu-speakers with a seminomadic pastoralist lifestyle^46^, and the Fwe, who have incorporated click consonants, typical of Khoisan idioms, into their language^47^. Previous studies showed admixture of these two groups with Khoisan neighbours^41^,^46^.

The second dimension (Fig. 3) separates Eastern African groups from the other populations. The most divergent group on this dimension is El Molo, a Kenyan Cushitic group known for its genetic isolation^48^. If a line was to be drawn between 0.2 and 0.3 of this dimension it would separate Eastern populations from Bantu groups, suggesting low intrusion of eastern African lineages into the expanding Bantu-speakers to the South, which otherwise would show a greater proximity to Eastern Africans. On the border of this hypothetical line lies Sudan and Luhya (LWK), from Eastern Africa, and three southern Bantu populations, Kunda (Zambia), Shona (Zimbabwe) and Nyaneka (Angola).

**Figure 4:**
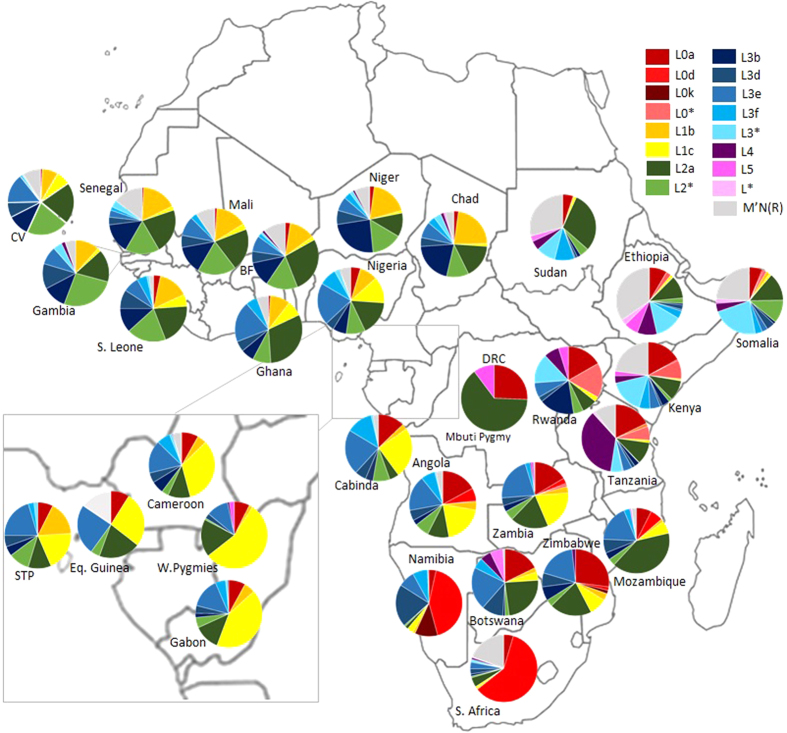
Haplogroup composition of sub-Saharan African countries. Population abbreviations: BF – Burkina Faso, CV – Cape Verde, S. Africa – South Africa, S. Leone – Sierra Leone, STP – São Tomé and Príncipe, W. Pygmies – Western Pygmies. The map was obtained from the website www.outline-world-map.com.

A detailed assessment of haplogroup composition was performed (Fig. 4 and S5). Nyaneka displays a haplogroup composition typical of Bantu populations and absence of Eastern lineages, but a fairly high frequency of L0a (~20%) (Fig. 4 and S5). In the second MDS (Supplementary Fig. 4), excluding L2a and L0a haplotypes, this proximity to Eastern Africa was no longer visible. Shona presents non-L lineages (~5%) and some typically eastern lineages, like L4, at very low frequencies (~3%) (Supplementary Fig. 5). Kunda harbours lineages labelled as L0*(~5%) at a frequency comparable to Eastern Africa (Fig. 3 and Supplementary Fig. 5). Overall, these three populations have almost an entirely Central African Bantu ancestry and there is little evidence of contact with Eastern populations.

On the other hand, the Luhya and Sudan are the eastern groups with the closest positions to southern Bantus. The Luhya, a Kenyan Bantu-speaking group, seems to display a dual Bantu/East African maternal ancestry. It harbours a high proportion of L3b (typical of Western Africa) and, on the other hand, L0a, L4 and L5 at frequencies comparable to Eastern Africa (Supplementary Fig. 5). This suggests Bantu genetic input into Eastern Africa (as expected since the Luhya are Bantu speakers) but not the opposite. The position of Sudan is essentially due to its high frequency of L2a (Fig. 2a and 4), since its proximity to Western and Bantu populations disappears when excluding L2 sequences from the analysis (Supplementary Fig. 4). L2a reaches a peak in Sudan (~30%), much higher than the average for Eastern Africa (~12%) and more similar to Western Africa (Supplementary Fig. 5). This proximity was probably established in the postglacial period, based on our phylogeographic analysis performed here.

## Discussion

We extensively analysed the phylogeography of major African mtDNA haplogroup L2. Following an origin in Western or Central Africa, L2 was involved at least in three movements out of Central Africa:

1. in the Pleistocene, at least 50 ka ago, documented by the emergence of L2a5 either in Eastern or Southern Africa whose arrival could match a period of climate change that could have also triggered the OOA migration and Eastern to Central African movements^12^,^44^.
2. in the postglacial period comprising the Late Pleistocene and Early Holocene (a period that witnessed major changes in climate and vegetation^6^,^10^), comprising mainly the expansion of L2a throughout Eastern Africa, in the opposite direction of L0a haplogroup^2^.
3. in the Late Holocene, L2 clades, mostly L2a clades, were deeply involved in the Bantu expansion to Southern Africa.

Although archaeological evidence suggests that the Bantu dispersals towards Southern Africa paused for a few hundred years at the Great Lakes region and some gene flow with Eastern populations into the Bantu arriving group scan therefore be expected before they expanded South, specific eastern mtDNA lineages (like L4, L5, L6 and several L3 sub-clades) are not detected at considerable frequencies in Southern Bantu-speaking populations. MtDNA haplogoups L0a and L2 were present in Central and Eastern Africa at least since the Early Holocene and they could have moved to the South from a dual source. However at whole mtDNA genome resolution, we could see that at least three quarters of L0a and L2 in the south were originated in Central Africa in the last 5 ka. This suggests a low maternal Eastern African ancestry in the Southern Bantu populations, confirmed by genetic distances on the HVS-I population level.

Y-chromosome evidence also indicates little gene flow in the paternal component between eastern populations and the Bantu migrants^49^. Uganda represents currently the major gap for mtDNA sampling and, since it lies in the transition between Nilotic and Niger-Kordofanian languages, it could provide valuable insights on the dynamics of Bantu and non-Bantu populations in the Great Lakes region. There is however Y-chromosome information for Uganda but only regarding Nilotes^49^,^50^, who present low proportion of shared haplotypes with Bantu neighbours, suggesting at least low gene flow from Bantu to Nilotic people. Genome-wide data suggests a shared component across most of Bantu populations^51^. A recent high resolution characterization of African populations supports this same figure^52^. At a likely estimation with six components (K = 6), the dominant Western/Central component prevails at Bantu-speaking populations in Southern Africa (admixed with the component found in Khoisan populations) and Eastern Africa, where it is admixed with an eastern component that was basically not detected in the south^52^. However, autosomal information from a typical Southeast African Bantu population is still unavailable and so it is difficult to assess an Eastern African input in the eastern Bantu route into Southern Africa at the genome-wide level.

The Bantu expansion is often placed within a group of linked theories proposing demographic expansions, associated with a specific language and triggered by the advent of agricultural practices called the Language-Farming dispersal hypothesis^16^, which includes the spread of Indo-European in Europe and Austronesian in Island Southeast Asia (ISEA) and the Pacific. While genetic patterns of current European and ISEA populations are difficult to reconcile with a demographic diffusion model associated with language and agricultural dispersal^9^,^53^, African Bantu-speaking populations display a genetic signature that allows linking them to a Central African origin. While genetic incorporation of autochthonous diversity into Bantu-speaking populations certainly occurred during the last two millennia throughout Africa^41^,^43^,^46^ the phylogeography of mtDNA haplogroups L2 and L0a as well as the population analyses performed here suggest that that incorporation was minimal during early Bantu expansion through the Eastern Route that continuously mimics the expansion of its Central African ancestral population, even when considering that Bantu-speakers were stationed in Eastern Africa for a few hundred years before their migration southwards.

Understanding worldwide population history is essential for studies on disease susceptibility, adaptation and pharmacogenetics and Africa as the cradle of modern humans and the most genetically diverse continent plays a central role in this genetic characterization^52^. Central Africa that, we hypothetically placed before as a likely point of origin of the modern humans^2^, was also the source for migrations in the Late Pleistocene/Early Holocene and more drastically a demic diffusion in the last few millennia that carried both signals of genetic adaptation developed in Central Africa as well as eventually recently developed pathogenic mutations^54^ throughout the continent.

## Methods

### Sampling, mtDNA sequencing and haplogroup affiliation

We targeted 44 samples representative of the diversity of haplogroup L2 in different African regions for complete mtDNA sequencing: four individuals from Ethiopia, 12 from Somalia and nine from Sudan (a total of 25 samples from Eastern Africa), 14 from Mozambique (Southeast Africa) and five from São Tomé and Príncipe (Western Africa). All the samples belonged to unrelated individuals who gave informed consent. Sudanese and Ethiopian samples were collected from emigrants in Dubai and Somali samples were from refugees in Yemen. Samples from Mozambique and São Tomé and Príncipe were collected locally, in Maputo and Príncipe Island, respectively. The work was approved by the Ethics Committee of the University of Porto (11/CEUP/2011).

We performed amplification and Sanger sequencing reactions as reported previously^55^. We compared sequences to rCRS^56^ using Geneious v.5.4^57^ and we manually checked and annotated polymorphisms according to the nomenclature in PhyloTree (Build 16, February 2014)^58^. We combined these sequences with published complete L2 mtDNA sequences for a total of 801 L2 complete sequences (Supplementary Table 3). For comparative purposes, we also performed a reanalysis of L0a phylogeny^2^ (based on published whole-mtDNA sequences), with a total 303 mtDNA genomes (Supplementary Table 4). We used MtDNA GenSyn software^59^ to convert sequences into haplotypes. Sequences are available at GenBank with accession numbers KR135841- KR135884.

### Phylogenetic reconstruction

Phylogeny was reconstructed based on a preliminary reduced-median network analysis with Network v.4.611^60^, which suggested a branching order that was manually constructed considering the frequency of each mutation as reported^23^ and the additional guidance of PhyloTree (Build 16).

In order to estimate the TMRCA of L2 and L0a internal clades, we used both rho (*ρ*) statistics and maximum likelihood (ML) analyses. In addition to the positions removed for phylogenetic reconstruction, we also excluded all indels for the following analysis, since this type of variation is not considered by the models used for age estimation. *ρ* statistics^61^ estimates the average of mutational steps from a given ancestral node to the tips of the phylogeny purely based on a given mutation rate, not including any evolutionary model. The mutation rate applied was one substitution every 3,624 years and corrected for purifying selection and the synonymous mutation rate was one substitution every 7,884 years^23^. Standard errors were estimated realistically^62^. We performed ML estimates of branch lengths using PAML 4^63^. We assumed the HKY85 mutation model as done previously^23^ with gamma-distributed rates (discrete distribution of 32 categories). We considered two partitions so as to differentiate the fast evolving HVS-I and HVS-II from the rest of the molecule.

Evidence of violation of the molecular clock was previously reported for African haplogroups, mostly L2^26^,^31^,^32^. We conducted the PAML analysis for this haplogroup both with and without a molecular clock and performed a likelihood ratio test, which indicated deviations to the molecular clock. In addition, we estimated ages of L2 internal nodes using BEAST v.1.8.0^64^ (100,000,000 interactions with a burn-in of 10,000,000 steps), applying both a strict and a relaxed molecular clock (which allows rate variation across lineages) and a mutation rate of 2.6186 × 10^−8^ substitutions per site per year (calculated previously for haplogroup L3^12^). We compared both analyses by calculating a Bayes factor, which showed very strong differences^65^, supporting the use of a relaxed clock for L2. However, when we compared calculated branch lengths in the Bayesian inference and ML they were extremely similar.

We assessed variations in the effective population size (Ne) associated to haplogroup L2 over time with Bayesian Skyline Plots (BSPs)^66^, also obtained with BEAST v.1.8.0 and visualized with Tracer v.1.6 ( http://beast.bio.ed.ac.uk/Tracer). Haplogroup L2 does not equate to population data, but a BSP applied to a specific lineage is expected to provide insights into the increments of the population associated with that lineage. This kind of approach has been performed before with complete mtDNA sequences for various haplogroups with satisfactory results^2^,^12^,^28^,^35^. We assumed a 25-year generation time^67^. We organized the samples in major monophyletic groups to resemble main subhaplogroups (L2a, L2a1, L2b, L2c, L2d and L2e), in order to guarantee a tree structure similar to our phylogenetic reconstruction and allow direct comparison among different analyses. Apart from the BSP for the entire sub-Saharan African dataset of complete L2 sequences, additional regional BSPs (Western/Central, Eastern and Southern Africa) were computed.

We additionally performed reduced-median network analysis^60^ of haplogroups L0a and L2 (based on HVS-I). We calculated founder ages of L2a main nodes in Eastern Africa considering the mutation rate previously calculated for HVS-I^33^ scaled to the size of the fragment considered (position 16090 to 16365).

### Frequency distribution maps and population comparisons

In order to visualize their geographic distribution within Africa, we constructed frequency distribution maps (based on HVS-I data) for major L2 subhaplogroups (L2a, L2b, L2d, L2e and L2*) with Surfer® v.8 (Golden Software) using Kriging algorithm. L2c is not distinguishable by HVS-I polymorphisms alone^18^, therefore we calculated its frequency as L2*. The dataset included 13910 HVS-I samples from 39 different countries (Supplementary Table 5). This dataset contains published HVS-I African sequences, plus the HVS-I segment of complete mtDNA sequences from 1000 Genomes^68^ and recent population studies^41^,^42^,^43^,^69^,^70^,^71^.

From the dataset used to compute the frequency distribution maps, we considered only populations with more than 30 individuals and with ethnic and/or linguistic information available for population-based analysis. However, the general populations that were sampled for the L2 phylogeography analysis (Ethiopia, Mozambique, São Tomé and Príncipe, Somalia and Sudan) were also included. Since the main goal was to infer the relationships between sub-Saharan groups (particularly between Bantu and Eastern groups), populations from North Africa, Pygmy and Khoisan groups were not included.

We computed genetic distances between pairs of populations (F_ST_) based on a 276 bp fragment of HVS-I with Arlequin v.3.5.1.3^72^ (10,000 permutations). The fragment considered corresponds to the smaller range common to all publications (from position 16090 to 16365). We represented relationships among populations by multidimensional scaling (MDS) plots (obtained with IBM® SPSS Statistics v.22), based on Slatkin’s linearized F_ST_^73^. We merged same ethnic groups from different countries and/or different studies, as well as different ethnic groups from the same country and we considered them as one unique population whenever F_ST_ was not significant in a preceding analysis. We always applied Bonferroni correction to p-values. The final MDS plot includes 55 populations (Supplementary Table 6), corresponding to 4880 individuals. In addition, we assessed genetic distances excluding L2a and L0a sequences and we constructed a second MDS plot, based on a total of 3323 individuals. We assessed mtDNA haplogroup composition in Western, Central, Eastern and Southern Africa (both by region and by country) (Supplementary Table 7).

## Additional Information

**How to cite this article**: Silva, M. *et al.* 60,000 years of interactions between Central and Eastern Africa documented by major African mitochondrial haplogroup L2. *Sci. Rep.* **5**, 12526; doi: 10.1038/srep12526 (2015).

## Supplementary Material

Supplementary Information

Supplementary Tables

## Figures and Tables

**Table 1:** Age estimates of L2 and L0a clades mentioned in the text.

**Clade**	**Bayesian relaxed clock**	**ML whole mtDNA**	***ρ*****whole mtDNA**	***ρ*** **synonymous age**
L2	78600 [64900–92400]	99100 [83000–115500]	68800 [52200–86100]	76400 [46600–106200]
L2a’b’c’d	73700 [60500–87400]	93500 [78000–109300]	57200 [38500–76800]	77900 [41100–114700]
L2a	66100 [52700–79200]	84400 [67000–102200]	50100 [33100–68000]	70500 [35600–105400]
L2a1-4	45500 [34800–56600]	50700 [34700–67500]	28300 [20600–36300]	32400 [18500–46300]
L2a1	29600 [23400–36100]	26500 [17900–35500]	18900 [12000–26100]	28700 [11100–46200]
L2a1 + 143	23300 [19300–28000]	24800 [17500–32200]	23200 [18000–28500]	21000 [15900–26000]
L2a1a	8700 [6200–11000]	6200 [3800–8800]	5400 [3300–7600]	5900 [2000–9800]
L2a1a2a1a	8100 [6300–10300]	5300 [3200–7500]	6800 [3200–10400]	6600 [2500–10600]
L2a1b	14000 [11800–16300]	11400 [7100–15900]	14100 [6400–22100]	11000 [400–21500]
L2a1b1a	9400 [7300–11500]	6900 [4500–9300]	5000 [2800–7300]	3200 [600–5700]
L2a1c	17300 [14100–20500]	17000 [12600–21500]	17400 [13300–21500]	18000 [12100–24000]
L2a1d	15300 [12200–18900]	17600 [12200–23100]	22200 [12300–32500]	22400 [6200–38600]
L2a1d1	9900 [6500–13800]	10600 [5700–15700]	15500 [7700–23500]	19700 [4300–35200]
L2a1d2	13000 [10300–16000]	15800 [10100–21600]	17200 [6100–28900]	11800 [0–25500]
L2a1d2a	7700 [5800–10600]	3700 [1700–5700]	3500 [1800–5200]	3900 [800–7100]
L2a1e	13300 [9400–17100]	13300 [6700–20100]	13400 [6100–20900]	22300 [5800–38800]
L2a1f	18300 [13600–22600]	7500 [5100–9900]	7700 [5200–10300]	7000 [4000–10100]
L2a1g	6000 [2300–10700]	3700 [0–8600]	3900 [0–8400]	3900 [0–11700]
L2a1h	13300 [9200–17400]	14400 [6400–22800]	16900 [6200–28200]	23700 [2500–44800]
L2a1i	11700 [8900–14000]	9300 [5600–13100]	9400 [5100–13800]	9400 [2600–16100]
L2a1j	7600 [4500–11900]	7000 [0–16900]	6100 [1000–11400]	5300 [0–12500]
L2a1k	8800 [4900–13500]	9700 [3400–16300]	10600 [3200–18400]	7900 [0–18800]
L2a1l	12500 [10600–14100]	11000 [6800–15400]	11300 [16200–65600]	10000 [1100–18900]
L2a1l2a	6600 [4400–8800]	1500 [0–3600]	1800 [0–4300]	—
L2a1m	7700 [4900–10700]	6500 [2400–10800]	5700 [2000–9600]	9500 [800–18200]
L2a1q	6100 [3500]	2900 [0–6800]	1700 [0–4100]	2600 [0–7800]
L2a1r	8500 [5800–11200]	7300 [1500–13400]	9000 [2600–15600]	6300 [0–18700]
L2a2'3'4	32300 [22600–41200]	36500 [25200–48400]	37100 [25400–49300]	42800 [24200–61500]
L2a4	23600 [14800–32100]	27600 [17000–38600]	23700 [12300–35700]	42400 [15000–69800]
L2a4a	9900 [5800–14100]	6100 [1600–10600]	4800 [1300–8400]	3900 [0–9700]
L2a4b	10700 [5600–15800]	12500 [4500–21000]	13400 [5000–22200]	15800 [300–31200]
L2a5	46000 [32100–59200]	56200 [41300–71700]	50000 [34000–66800]	57400 [28600–86300]
L2b’c’d	65100 [52400–78700]	86700 [10400–70500]	79800 [10100–59300]	50500 [30100–70800]
L2b’c	49700 [37300–61000]	65300 [49700–81300]	59300 [43500–75800]	39200 [22900–55500]
L2b	24500 [19300–30700]	26000 [20300–31900]	27600 [19000–36600]	30900 [17000–44800]
L2b1a3	9800 [6900–12700]	5500 [2900–8100]	5200 [2400–8100]	7200 [600–13800]
L2b2a	7700 [4800–10800]	6100 [1900–10500]	4700 [1600–7800]	4700 [0–10100]
L2c	18300 [14500–22700]	17200 [13800–20600]	18600 [14300–23000]	15500 [10900–20100]
L2c2a1	7800 [5700–9700]	5900 [2000–10000]	8400 [1400–15800]	3200 [100–6200]
L2c2b1b	5000 [2900–7300]	2500 [0–5500]	2600 [0–5600]	2600 [0–7800]
L2d	16200 [12600–20400]	19000 [13200–24900]	16500 [9700–23500]	18600 [7300–29800]
L2e	34300 [24200–44900]	38900 [28400–49600]	28600 [20100–37400]	37200 [20700–53600]
L2e1a1a	5300 [2700–9000]	1800 [0–4200]	1700 [0–4100]	—
L0a	—	59200 [41000–78200]	38500 [27700–49700]	37700 [20700–54800]
L0a1a2	—	10500 [7700–13400]	7800 [5500–10100]	6300 [4100–8500]
L0a1b1a	—	6700 [3600–9900]	6300 [2000–10800]	3600 [1300–5800]
L0a2a2a	—	7000 [4700–9300]	5500 [3400–7700]	5300 [1400–9200]

Underlined branches are newly labelled in this study.
