# Supplementary material for: 60,000 years of interactions between Central and Eastern Africa documented by major African mitochondrial haplogroup L2
Source: Sci Rep. 2015 Jul 27;5:12526. doi: 10.1038/srep12526 (PMC4515592; doi:10.1038/srep12526)
Supplement: Supplementary Information [file srep12526-s1.pdf]

60,000 years of interactions between Central and Eastern Africa documented by major African mitochondrial haplogroup L2

Marina Silva, Farida Alshamali, Paula Silva, Carla Carrilho, Flávio Mandlate, Maria Jesus Trovoada, Viktor Černý, Luísa Pereira, Pedro Soares

Supplementary Fig. 1. Comparison between ML and Bayesian age estimates. Correlation between ML and Bayesian branch lengths, in nucleotides **(a)**. Variations in the ratio between ML and Bayesian node age estimates compared to the ML age of the branches, in years **(b)**.

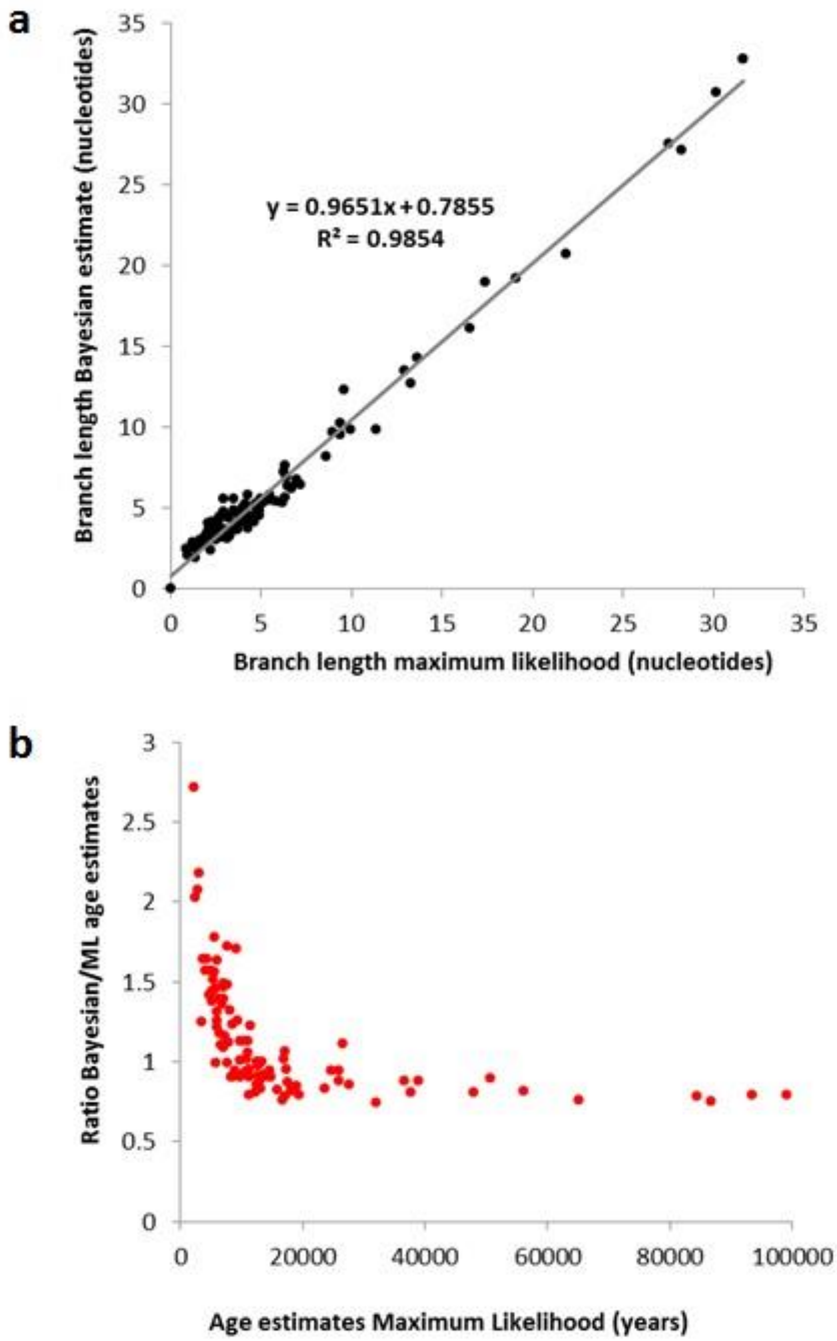

Supplementary Fig. 2. BSPs indicating the median of the Ne associated to haplogroup L2 through time. BSP based on all L2 complete sequences from sub-Saharan Africa **(a)**, with a maximum time (~78 ka) corresponding to the mean posterior estimate of the genealogy root-height. BSPs for different African regions: Western/Central Africa **(b)**, Eastern Africa/Near East/Arabia Peninsula **(c)** and Southern Africa **(d)**.

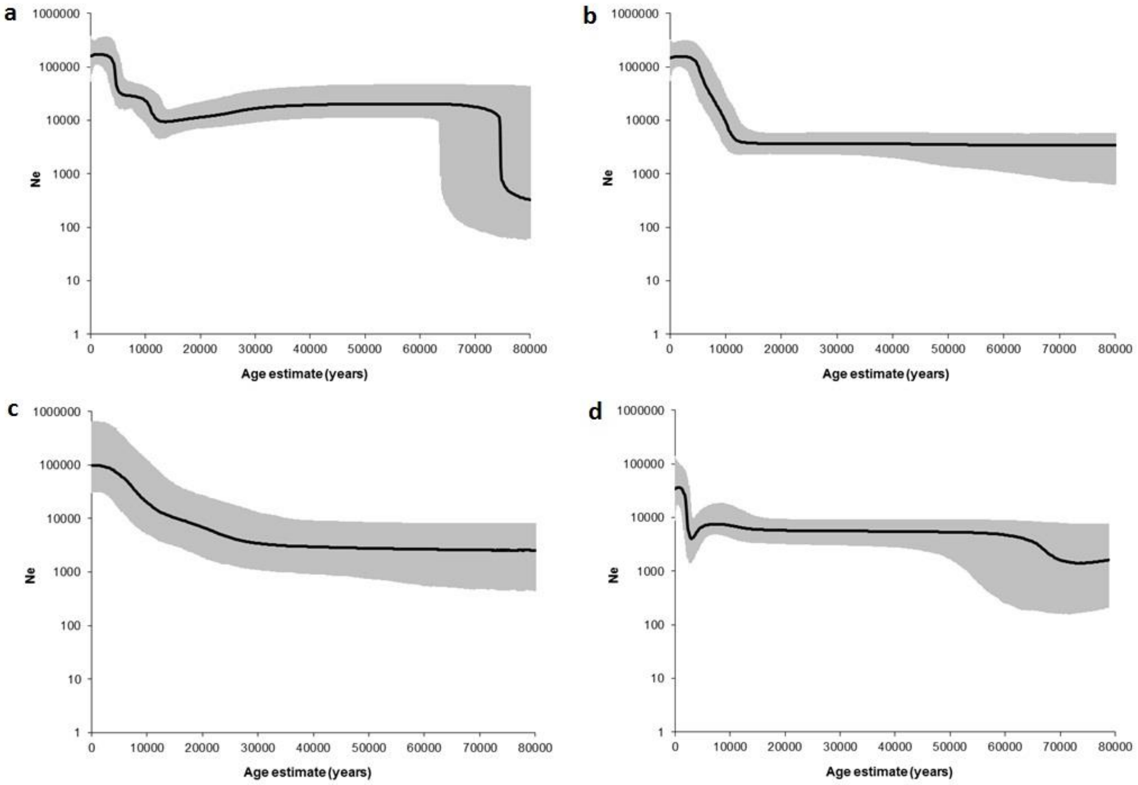

Supplementary Fig. 3. Graphs of population increments for Western/Central Africa (WA/CA), Eastern Africa/Near East/ Arabian Peninsula (EA/Arabia) and Southern Africa

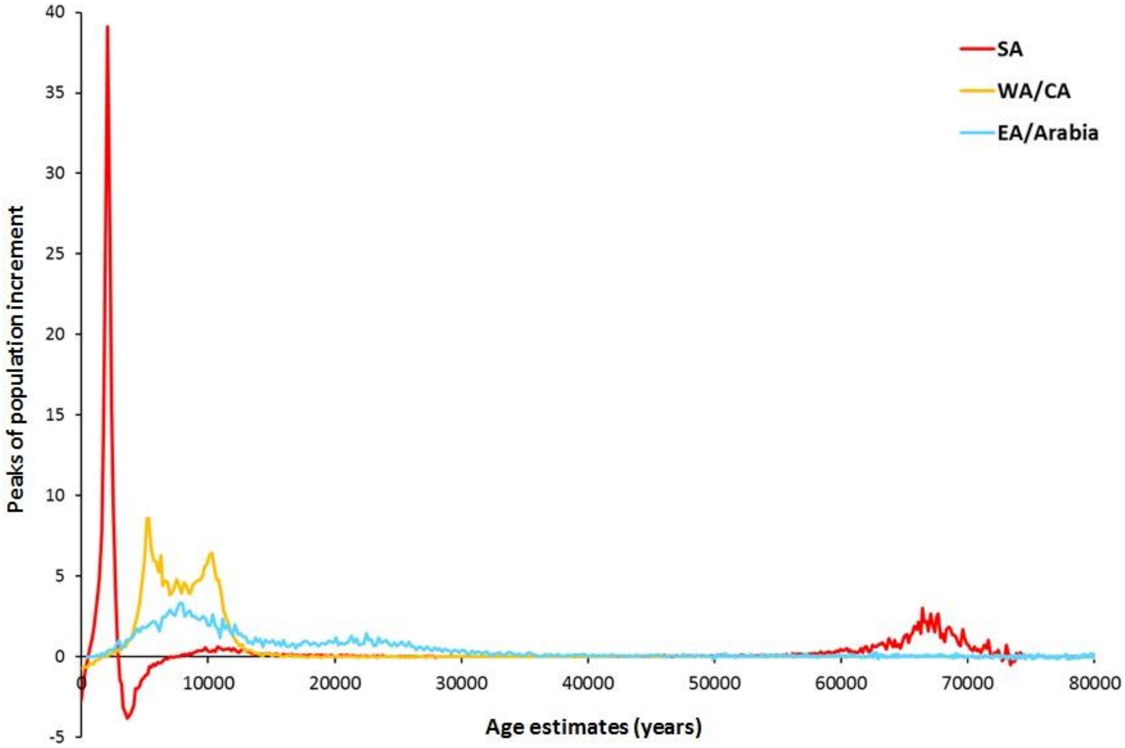

Supplementary Fig. 4. MDS plot based on Sltkin's linearized F_{ST}, excluding L2a and L0a sequences. Colour code: WA – Western Africa, CA – Central Africa, EA – Eastern Africa, SA – Southern Africa.

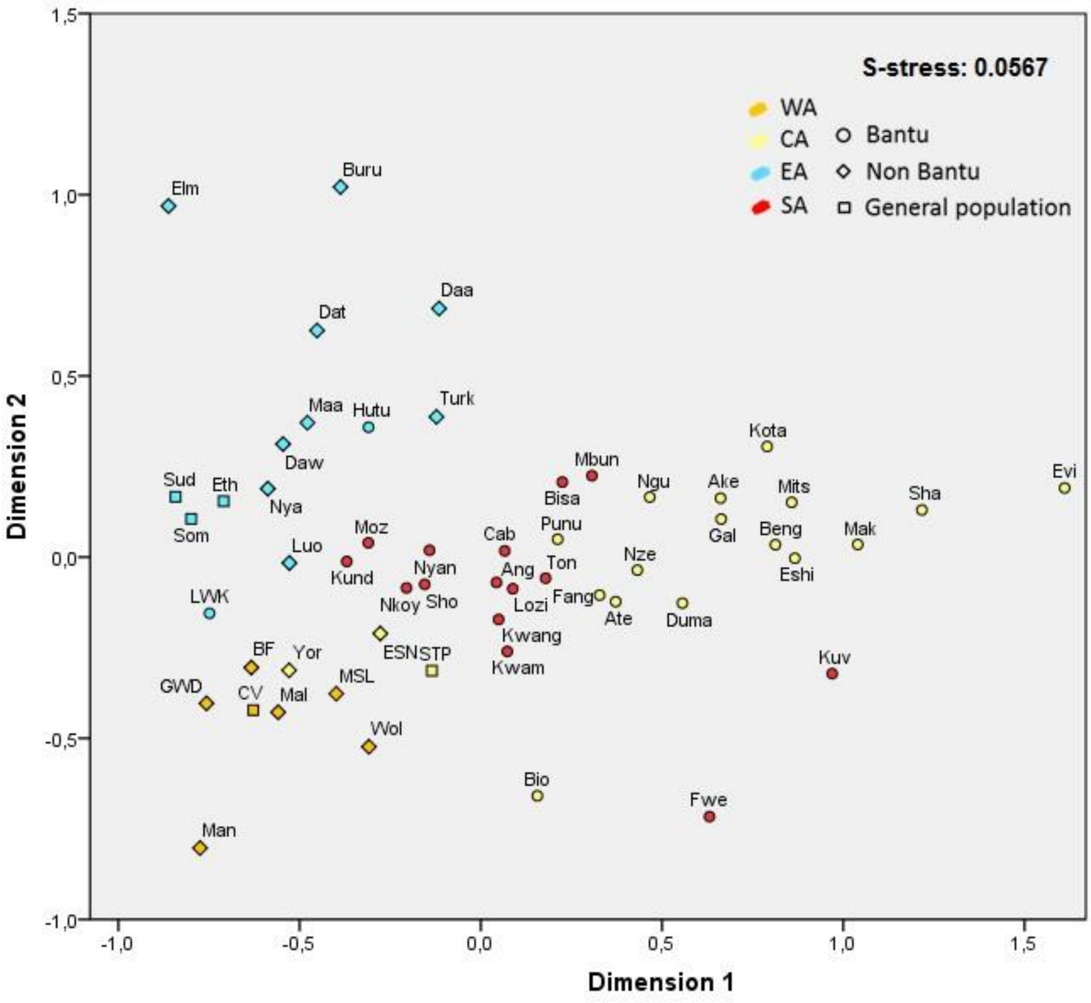

Supplementary Fig. 5. Haplogroup composition of sub-Saharan African regions (WA – Western Africa, CA – Central Africa, EA – Eastern Africa, SA – Southern Africa), compared to Sudan, Luhya (LWK), Kunda, Shona and Nyaneka. The map was obtained from the website www.outline-world-map.com.

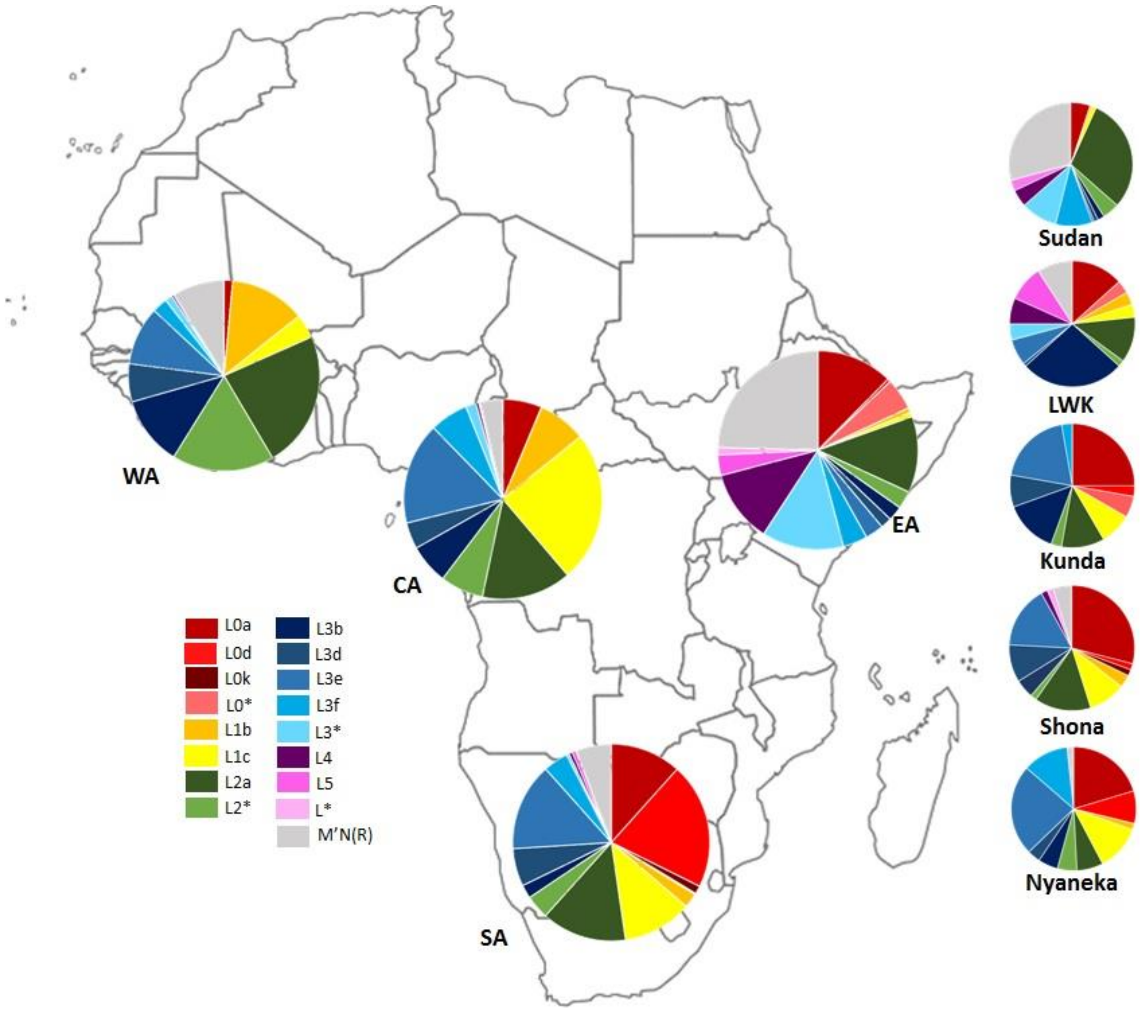

Excel file:

Supplementary Table 1. Phylogenetic tree of mtDNA haplogroup L2. Most parsimonious phylogeny of mtDNA haplogroup L2, based on a total of 801 L2 complete mitogenomes. Polymorphic positions annotated along the branches (mutations in relation to an ancestral state): uppercase for transitions and lowercase for transversions, back mutations indicated with an exclamation mark (double back mutations indicated with two exclamation marks), insertions and deletions indicated by a dot and the letter "d", respectively (shaded in grey; not considered for age estimations). New branch labels in red and shaded in yellow, braches with major alterations in comparison to PhyloTree Build 16 in rose. More detailed information on the samples and/or references on Supplementary Table 3.

Supplementary Table 2. Phylogenetic tree of mtDNA haplogroup L0a. Most parsimonious phylogeny of mtDNA haplogroup L0a, based on a total of 303 L0a complete mitogenomes. Polymorphic positions annotated along the branches (mutations in relation to an ancestral state): uppercase for transitions and lowercase for transversions, back mutations indicated with an exclamation mark (double back mutations indicated with two exclamation marks), insertions and deletions indicated by a dot and the letter "d", respectively (shaded in grey; not considered for age estimations). More detailed information on the samples and /or references on Supplementary Table 4.

Supplementary Table 3. List of 801 complete L2 sequences used for phylogenetic reconstruction and phylogeography analysis. Codes for regions: AM – America, CA – Central Africa, EA – Eastern Africa, NA – Northern Africa, NE/AP – Near East/Arabian Peninsula, SA – Southern Africa, WA – Western Africa, EUR – Europe. Abbreviations: G. Bissau – Guinea-Bissau, S. Africa – South Africa, STP – São Tomé and Príncipe, S. Arabia – Saudi Arabia, USA – United States of America. Additional information on ethnicity or geographical location indicated whenever provided by the authors. Branches labelled in this study are underlined.

Sample	Haplogroup	Region	Country / Ethnic group	Additional information	Reference
ETH31	L2a1+143	EA	Ethiopia	-	Present study
EU092671	L2a1+143+16189	NA	Morocco	Jew	[1]
HG03121	L2a1+143+16189	CA	Nigeria	Esan	[2]
JQ044837	L2a1+143+16189	WA	Burkina Faso	-	[3]
SUD43	L2a1+143+16189	EA	Sudan	-	Present study
SUD12	L2a1+143+16189	EA	Sudan	-	Present study
ETH5	L2a1+143+16189	EA	Ethiopia	-	Present study
SOM76	L2a1+143+16189	EA	Somalia	-	Present study
EU092679	L2a1+143+16189+16192	AP / NE	Israel	Palestinian	[1]
EU092782	L2a1+143+16189+16192	AP / NE	Oman	-	[1]
EU092793	L2a1+143+16189+16192	AP / NE	Yemen	-	[1]
JQ702430	L2a1+143+16189+16192	AM	USA	-	[1]
EU092806	L2a1+143+16189+16192	NA	Morocco	-	[1]
Howell434	L2a1+143+16189+16192	AM	USA	-	[4]
EU092659	L2a1+143+16189+16192	AP / NE	Israel	Druze	[1]
FJ460527	L2a1+143+16189+16192	NA	Tunisia	-	[5]
EU092674	L2a1+143+16189+16192	EA	Ethiopia	Jew	[1]
HG03342	L2a1+143+16189+16192	CA	Nigeria	Esan	[2]
ETH23	L2a1+143+16189+16192	EA	Ethiopia	-	Present study
EU092823	L2a1+143+16189+16192	NA	Libya	-	[1]
EU092658	L2a1+143+16189+16192	AP / NE	Israel	Bedouin	[1]
EU597491	L2a1+143+16189+16192	AP / NE	Israel	Bedouin	[6]
FJ460520	L2a1+143+16189+16309	NA	Tunisia	-	[5]
JQ044822	L2a1+143+16189+16309	WA	Burkina Faso	-	[3]
JQ044839	L2a1+143+16189+16309	WA	Burkina Faso	-	[3]
HG03485	L2a1+143+16189+16309	WA	Sierra Leone	Mende	[2]
JQ045062	L2a1+16189	WA	Burkina Faso	-	[3]
HG03028	L2a1+16189	WA	Gambia	-	[2]
Howell195	L2a1a	AM	USA	-	[4]
Howell382	L2a1a	AM	USA	-	[4]
Howell577	L2a1a	AM	USA	-	[4]
EU092916	L2a1a	EA	Kenya	-	[4]
DQ304926	L2a1a	AM	USA	-	[7]
JQ044922	L2a1a	WA	Burkina Faso	-	[3]
DQ304927	L2a1a	AM	USA	-	[7]
HG03114	L2a1a	CA	Nigeria	Esan	[2]
DQ304928	L2a1a	AM	USA	-	[7]
HG02588	L2a1a	WA	Gambia	-	[2]
DQ304924	L2a1a	AM	USA	-	[7]

Supplementary Table 3. (continued)

DQ304932	L2a1a	AM	USA	-	[7]
HG02501	L2a1a	AM	Barbados	African Caribbean	[2]
JQ044942	L2a1a	WA	Burkina Faso	-	[3]
JQ044885	L2a1a	WA	Burkina Faso	-	[3]
JQ044961	L2a1a	WA	Burkina Faso	-	[3]
SUD56	L2a1a	EA	Sudan	-	Present study
HG02852	L2a1a	WA	Gambia	-	[2]
HG02860	L2a1a	WA	Gambia	-	[2]
JQ044927	L2a1a	WA	Burkina Faso	-	[3]
JQ044983	L2a1a	WA	Burkina Faso	-	[3]
JQ044992	L2a1a	WA	Burkina Faso	-	[3]
JQ705087	L2a1a	-	unknown	-	[8]
JQ044911	L2a1a	WA	Burkina Faso	-	[3]
JQ044818	L2a1a	WA	Burkina Faso	-	[3]
HG03563	L2a1a	WA	Sierra Leone	Mende	[2]
KJ185692	L2a1a	SA	Zambia	Mbunda	[9]
KJ185688	L2a1a	SA	Zambia	Mbunda	[9]
MOZ326	L2a1a	SA	Mozambique	-	Present study
MOZ33	L2a1a	SA	Mozambique	-	Present study
SOM68	L2a1a	EA	Somalia	-	Present study
Howell162	L2a1a1	AM	USA	-	[4]
Howell156	L2a1a1	AM	USA	-	[4]
Howell571	L2a1a1	AM	USA	-	[4]
DQ304925	L2a1a1	AM	USA	-	[7]
DQ304933	L2a1a1	AM	USA	-	[7]
JQ706014	L2a1a1	-	unknown	-	[8]
JQ045001	L2a1a1	WA	Burkina Faso	-	[3]
JQ044977	L2a1a1	WA	Burkina Faso	-	[3]
JQ045005	L2a1a1	WA	Burkina Faso	-	[3]
JQ045020	L2a1a1	WA	Burkina Faso	-	[3]
JQ045066	L2a1a1	WA	Burkina Faso	-	[3]
JQ045074	L2a1a1	WA	Burkina Faso	-	[3]
KJ185589	L2a1a1	SA	Zambia	Lozi	[9]
KJ185982	L2a1a1	SA	Zambia	Lunda	[9]
KJ185827	L2a1a1	SA	Angola	Ovimbundu	[9]
HG03460	L2a1a1	WA	Sierra Leone	Mende	[2]
HG03578	L2a1a1	WA	Sierra Leone	Mende	[2]
HG02922	L2a1a1	CA	Nigeria	Esan	[2]
HG03410	L2a1a1	WA	Sierra Leone	Mende	[2]
HG03548	L2a1a1	WA	Sierra Leone	Mende	[2]
HQ425645	L2a1a2	AM	USA	-	Family Tree
HG01363	L2a1a2	AM	Colombia	Medellín	[2]
JQ044987	L2a1a2	WA	Burkina Faso	-	[3]
HG03123	L2a1a2	CA	Nigeria	Esan	[2]
KC622072	L2a1a2	SA	Botswana	Kalanga	[10]
JQ044997	L2a1a2	WA	Burkina Faso	-	[3]

Supplementary Table 3. (continued)

JQ044809	L2a1a2	WA	Burkina Faso	-	[3]
JQ044812	L2a1a2	WA	Burkina Faso	-	[3]
JQ044952	L2a1a2	WA	Burkina Faso	-	[3]
JQ045076	L2a1a2	WA	Burkina Faso	-	[3]
JQ045027	L2a1a2	WA	Burkina Faso	-	[3]
JQ044879	L2a1a2	WA	Burkina Faso	-	[3]
JQ044884	L2a1a2	WA	Burkina Faso	-	[3]
JQ044945	L2a1a2	WA	Burkina Faso	-	[3]
JQ044951	L2a1a2	WA	Burkina Faso	-	[3]
JQ045048	L2a1a2	WA	Burkina Faso	-	[3]
Howell570	L2a1a2	AM	USA	-	[4]
DQ304971	L2a1a2	AM	USA	-	[7]
KC622070	L2a1a2	SA	Botswana	Tswana	[10]
KJ185440	L2a1a2	SA	Zambia	Luvale	[9]
MOZ88	L2a1a2	SA	Mozambique	-	Present Study
JN214449	L2a1a2	EUR	Italy	Umbria (Centre)	[11]
JN214457	L2a1a2	EUR	Italy	Marche (Centre)	[11]
Howell569	L2a1a2	AM	USA	-	[4]
Howell380	L2a1a2	AM	USA	-	[4]
DQ304977	L2a1a2	AM	USA	-	[7]
DQ304968	L2a1a2	AM	USA	-	[7]
AF346977	L2a1a2	-	unknown	Effik	[12]
EU092691	L2a1a2	SA	Mozambique	Tswa	[1]
EU092804	L2a1a2	AP / NE	Yemen	-	[1]
EU092933	L2a1a2	AS	Pakistan	Makrani	[1]
JQ705250	L2a1a2	-	unknown	-	[8]
KC622069	L2a1a2	SA	Botswana	Tswana	[10]
KJ185769	L2a1a2	SA	Angola	Nyaneka	[9]
JX303760	L2a1a2	SA	Zambia	-	[13]
KJ185896	L2a1a2	SA	Zambia	Kwangwa	[9]
KJ185768	L2a1a2	SA	Angola	Nyaneka	[9]
KJ185829	L2a1a2	SA	Angola	Ovimbundu	[9]
KJ185934	L2a1a2	SA	Zambia	Makoma	[9]
MOZ142	L2a1a2	SA	Mozambique	-	Present study
MOZ99	L2a1a2	SA	Mozambique	-	Present study
SOM72	L2a1a2	EA	Somalia	-	Present study
DQ304975	L2a1a2a	AM	USA	-	[7]
JQ044968	L2a1a2a1	WA	Burkina Faso	-	[3]
JQ044845	L2a1a2a1	WA	Burkina Faso	-	[3]
MOZ36	L2a1a2a1	SA	Mozambique	-	Present study
DQ304969	L2a1a2a1a	AM	USA	-	[7]
DQ304970	L2a1a2a1a	AM	USA	-	[7]
KJ185973	L2a1a2a1a	SA	Zambia	Kaonde	[9]
KJ185733	L2a1a2a1a	SA	Zambia	Nkoya	[9]
KJ185732	L2a1a2a1a	SA	Zambia	Nkoya	[9]
DQ304976	L2a1a2a1a	AM	USA	-	[7]

Supplementary Table 3. (continued)

DQ304974	L2a1a2a1a	AM	USA	-	[7]
Howell142	L2a1a2a1a	AM	USA	-	[4]
EU092829	L2a1a2a1a	NA	Tunisia	-	[1]
JQ045017	L2a1a2a1a	WA	Burkina Faso	-	[3]
JQ705055	L2a1a2a1a	SA	Mozambique	-	[8]
KJ185826	L2a1a2a1a	SA	Angola	Ovimbundu	[9]
KJ185679	L2a1a2a1a	SA	Zambia	Mbunda	[9]
KJ185824	L2a1a2a1a	SA	Angola	Ovimbundu	[9]
MOZ317	L2a1a2a1a	SA	Mozambique	-	Present study
EU092872	L2a1a2a1a	SA	S. Africa	SEB	[1]
KJ185997	L2a1a2a1a	SA	Zambia	Ngoni	[9]
Howell562	L2a1a2a1a	AM	USA	-	[4]
HG02941	L2a1a2a1a	CA	Nigeria	Esan	[2]
EU092778	L2a1a2a1a	NA	Egypt	-	[1]
KJ185455	L2a1a2a1a	SA	Zambia	Lenje	[9]
DQ304972	L2a1a2b	AM	USA	-	[7]
DQ304973	L2a1a2b	AM	USA	-	[7]
Howell199	L2a1a2b	AM	USA	-	[4]
HG02439	L2a1a2b	AM	Barbados	African Caribbean	[2]
JQ045088	L2a1a2b	WA	Yoruba	Yoruba	[3]
JQ045095	L2a1a2b	WA	Yoruba	Yoruba	[3]
HG02676	L2a1a3	WA	Gambia	-	[2]
HG03539	L2a1a3	WA	Gambia	-	[2]
HG02870	L2a1a3	WA	Gambia	-	[2]
JQ044892	L2a1a3	WA	Burkina Faso	-	[3]
JQ045015	L2a1a3	WA	Burkina Faso	-	[3]
JQ044877	L2a1a3	WA	Burkina Faso	-	[3]
EU092711	L2a1a3	EUR	Portugal	Portuguese	[1]
EU092890	L2a1a3a	CA	Chad	Sara	[1]
EU092905	L2a1a3a	CA	Chad	Sara	[1]
JQ044813	L2a1a3a	WA	Burkina Faso	-	[3]
JQ705529	L2a1a3a	-	unknown	-	[8]
JQ044918	L2a1a3b	WA	Burkina Faso	-	[3]
JQ044819	L2a1a3b	WA	Burkina Faso	-	[3]
DQ304948	L2a1a3b	AM	USA	-	[7]
HM771168	L2a1a3c	CA	Pygmy	Pygmy	[14]
HG03169	L2a1a3c	CA	Nigeria	Esan	[2]
KJ185771	L2a1a3c	SA	Angola	Nyaneka	[9]
KC622184	L2a1a3c	SA	Namibia	Himba	[10]
STP99	L2a1a3c	WA	STP	-	Present study
KJ185770	L2a1a3c	SA	Angola	Nyaneka	[9]
JQ045101	L2a1a3c	WA	Yoruba	Yoruba	[3]
HG02977	L2a1a3c	CA	Nigeria	Esan	[2]
JQ044975	L2a1b	WA	Burkina Faso	-	[3]
JQ044841	L2a1b	WA	Burkina Faso	-	[3]
NA20274	L2a1b	AM	USA	-	[2]

Supplementary Table 3. (continued)

KJ185828	L2a1b	SA	Angola	Ovimbundu	[9]
EU092761	L2a1b	NA	Egypt	-	[1]
MOZ95	L2a1b1	SA	Mozambique	-	Present study
HG03132	L2a1b1	CA	Nigeria	Esan	[2]
HG02577	L2a1b1	AM	Barbados	African Caribbean	[2]
HG03202	L2a1b1	CA	Nigeria	Esan	[2]
MOZ64	L2a1b1a	SA	Mozambique	-	Present study
JX303858	L2a1b1a	SA	Zambia	Mbukushu	[13]
JX303761	L2a1b1a	SA	Zambia	Tonga	[13]
KJ185735	L2a1b1a	SA	Zambia	Nkoya	[9]
KJ185931	L2a1b1a	SA	Zambia	Makoma	[9]
KJ185825	L2a1b1a	SA	Angola	Ovimbundu	[9]
EU092690	L2a1b1a	SA	Mozambique	Chopi	[1]
EU092910	L2a1b1a	EA	Kenya	-	[1]
KC622066	L2a1b1a	SA	Botswana	Kalanga	[10]
MOZ318	L2a1b1a	SA	Mozambique	-	Present study
KJ185442	L2a1b1a	SA	Zambia	Luvale	[9]
JX303754	L2a1b1a	SA	Zambia	Tonga	[13]
KC622071	L2a1b1a	SA	Botswana	Kalanga	[10]
KJ185923	L2a1b1a	SA	Zambia	Luyana	[9]
JX303874	L2a1b1a	SA	Zambia	Tonga	[13]
JX303880	L2a1b1a	SA	Zambia	Totela	[13]
MOZ62	L2a1b1a	SA	Mozambique	-	Present study
MOZ45	L2a1b1a	SA	Mozambique	-	Present study
SOM13	L2a1b1a	EA	Somalia	-	Present study
SOM144	L2a1b1a	EA	Somalia	-	Present study
EU092705	L2a1b1a	SA	Mozambique	-	[1]
KC622057	L2a1b1a	SA	Botswana	Tswana	[10]
KJ185588	L2a1b1a	SA	Zambia	Lozi	[9]
KJ185452	L2a1b1a	SA	Zambia	Ila	[9]
KC622062	L2a1b1a	SA	Botswana	Tswana	[10]
KJ185587	L2a1b1a	SA	Zambia	Lozi	[9]
EU092919	L2a1b1a	AP / NE	Jordan	-	[1]
JQ044973	L2a1c	WA	Burkina Faso	-	[3]
DQ304949	L2a1c	AM	USA	-	[7]
DQ304944	L2a1c	AM	USA	-	[7]
EU092901	L2a1c	CA	Chad	Sara	[1]
HM771224	L2a1c	CA	Pygmy	-	[14]
EU092733	L2a1c	WA	G. Bissau	Balanata	[1]
EU092937	L2a1c	EA	Ethiopia	-	[1]
JN858955	L2a1c	CA	Cameroon	-	[11]
JQ702968	L2a1c	-	unknown	-	[8]
EU092683	L2a1c	AP / NE	Israel	Palestinian	[1]
KJ185486	L2a1c	SA	Angola	Ganguela	[9]
JQ045019	L2a1c	WA	Burkina Faso	-	[3]
JQ702307	L2a1c	-	unknown	-	[8]

Supplementary Table 3. (continued)

HG02808	L2a1c	WA	Gambia	-	[2]
JQ702503	L2a1c	AM	Mexico	-	[8]
HG02502	L2a1c	AM	Barbados	African Caribbean	[2]
HG02679	L2a1c	WA	Gambia	-	[2]
HG03078	L2a1c	WA	Sierra Leone	Mende	[2]
KJ185999	L2a1c	SA	Zambia	Nsenga	[9]
ETH48	L2a1c+16129	EA	Ethiopia	-	Present study
FJ460560	L2a1c1	NA	Tunisia	-	[5]
JQ044909	L2a1c1	WA	Burkina Faso	-	[3]
Howell561	L2a1c1	AM	USA	-	[4]
JQ044860	L2a1c1	WA	Burkina Faso	-	[3]
JQ044974	L2a1c1	WA	Burkina Faso	-	[3]
JQ045124	L2a1c1	WA	Mandenka	Mandenka	[3]
JX303852	L2a1c1	SA	Zambia	Subiya	[13]
KJ185731	L2a1c1	SA	Zambia	Nkoya	[9]
KJ185595	L2a1c1	SA	Zambia	Lozi	[9]
JQ045064	L2a1c1	WA	Burkina Faso	-	[3]
JQ705046	L2a1c1a	WA	Ghana	-	[8]
JQ044799	L2a1c1a	WA	Burkina Faso	-	[3]
Howell165	L2a1c1a	AM	USA	-	[4]
JQ702261	L2a1c1a	-	unknown	-	[8]
EU092954	L2a1c1a	EA	Ethiopia	-	[1]
JX524225	L2a1c1a	AM	Brazil	-	Family Tree
HG03117	L2a1c1a	CA	Nigeria	Esan	[2]
JQ044962	L2a1c2	WA	Burkina Faso	-	[3]
JQ045108	L2a1c2	WA	Mandenka	Mandenka	[3]
JQ044935	L2a1c2	WA	Burkina Faso	-	[3]
JQ044844	L2a1c2	WA	Burkina Faso	-	[3]
JQ044802	L2a1c2	WA	Burkina Faso	-	[3]
HG02819	L2a1c2	WA	Gambia	-	[2]
JQ044998	L2a1c2	WA	Burkina Faso	-	[3]
JQ045024	L2a1c2	WA	Burkina Faso	-	[3]
JQ044828	L2a1c2a	WA	Burkina Faso	-	[3]
JQ044924	L2a1c2a	WA	Burkina Faso	-	[3]
HM771169	L2a1c2a	CA	Pygmy	-	[14]
Tor65(#27)	L2a1c2a	AM	Dominica	-	[16]
JQ044872	L2a1c2a	WA	Burkina Faso	-	[3]
JQ044969	L2a1c2a	WA	Burkina Faso	-	[3]
JQ045025	L2a1c2a	WA	Burkina Faso	-	[3]
HG03449	L2a1c2a	WA	Sierra Leone	Mende	[2]
EU200762	L2a1c3	EUR	Slovenia	-	[17]
EU092663	L2a1c3	AP / NE	Israel	Bedouin	[1]
JN214440	L2a1c3	EUR	Spain	Galicia	[11]
HG03557	L2a1c3	WA	Sierra Leone	Mende	[2]
HG03571	L2a1c3	WA	Sierra Leone	Mende	[2]
JQ705145	L2a1c3	AM	USA	-	[8]

Supplementary Table 3. (continued)

HG02861	L2a1c3	WA	Gambia	-	[2]
HG02895	L2a1c3	WA	Gambia	-	[2]
EU092720	L2a1c3	WA	G. Bissau	FulaForro	[1]
JQ044996	L2a1c3	WA	Burkina Faso	-	[3]
JQ045063	L2a1c3	WA	Burkina Faso	-	[3]
HG02851	L2a1c3	WA	Gambia	-	[2]
JQ045122	L2a1c3	WA	Mandenka	Mandenka	[3]
JQ045110	L2a1c3	WA	Mandenka	Mandenka	[3]
JQ045116	L2a1c3	WA	Mandenka	Mandenka	[3]
HG03088	L2a1c3	WA	Sierra Leone	Mende	[2]
JN214432	L2a1c4	EUR	Spain	Andalusia	[11]
DQ304942	L2a1c4a	AM	USA	-	[7]
DQ304943	L2a1c4a	AM	USA	-	[7]
JQ044833	L2a1c4a	WA	Burkina Faso	-	[3]
JQ044957	L2a1c4a	WA	Burkina Faso	-	[3]
JQ045014	L2a1c4a	WA	Burkina Faso	-	[3]
JQ045035	L2a1c4a	WA	Burkina Faso	-	[3]
JQ045045	L2a1c4a	WA	Burkina Faso	-	[3]
JQ045067	L2a1c4a	WA	Burkina Faso	-	[3]
JQ701926	L2a1c4a	-	unknown	-	[8]
FJ460549	L2a1c4a	NA	Tunisia	-	[5]
DQ304951	L2a1c4a1	AM	USA	-	[7]
DQ304950	L2a1c4a1	AM	USA	-	[7]
KJ185487	L2a1c4a1	SA	Angola	Ganguela	[9]
KJ185488	L2a1c4a1	SA	Angola	Ganguela	[9]
Howell576	L2a1c5	AM	USA	-	[4]
JQ705001	L2a1c5	-	unknown	-	[8]
HM771225	L2a1c5	CA	Pygmy	-	[14]
HG02546	L2a1c5	AM	Barbados	African Caribbean	[2]
JQ045082	L2a1c5	WA	Yoruba	Yoruba	[3]
JQ045098	L2a1c5	WA	Yoruba	Yoruba	[3]
EU935443	L2a1c5	NA	Egypt	el-Hayez oasis	[18]
HG02455	L2a1c5	AM	Barbados	African Caribbean	[2]
HG02549	L2a1c5	AM	Barbados	African Caribbean	[2]
JX303749	L2a1c5	SA	Zambia	Totela	[13]
KJ185585	L2a1c5	SA	Zambia	Lozi	[9]
KJ185584	L2a1c5	SA	Zambia	Lozi	[9]
KJ185468	L2a1c5	SA	Zambia	Tonga	[9]
EF177417	L2a1c6	EUR	Portugal	-	[15]
JQ702659	L2a1c6	EUR	France	-	[8]
JN214436	L2a1c6	EUR	Spain	Galicia	[11]
NA20903	L2a1d	AM	USA	Gujarati Indian	[2]
EU092939	L2a1d1	EA	Ethiopia	-	[1]
SOM01	L2a1d1	EA	Somalia	-	Present study
EU092765	L2a1d1	NA	Egypt	-	[1]
EU092927	L2a1d1	NA	Egypt	-	[1]

Supplementary Table 3. (continued)

SUD54	L2a1d1	EA	Sudan	-	Present study
KJ185403	L2a1d2	SA	Zambia	Bemba	[9]
JN858956	L2a1d2	WA	Benin	-	[11]
JX303806	L2a1d2a	SA	Zambia	Kwamashi	[13]
JX303862	L2a1d2a	SA	Zambia	Mbukushu	[13]
JX303873	L2a1d2a	SA	Zambia	Tonga	[13]
KJ185686	L2a1d2a	SA	Zambia	Mbunda	[9]
KJ185591	L2a1d2a	SA	Zambia	Lozi	[9]
JX303795	L2a1d2a	SA	Zambia	Totela	[13]
JX303825	L2a1d2a	SA	Zambia	Mbukushu	[13]
JX303838	L2a1d2a	SA	Zambia	Mbukushu	[13]
KC622074	L2a1d2a	SA	Botswana	Tswana	[10]
KJ185459	L2a1d2a	SA	Zambia	Tokaleya	[9]
KJ185998	L2a1d2a	SA	Zambia	Nsenga	[9]
KJ185691	L2a1d2a	SA	Zambia	Mbunda	[9]
KJ185428	L2a1d2a	SA	Zambia	Luchazi	[9]
KJ185689	L2a1d2a	SA	Zambia	Mbunda	[9]
KJ185590	L2a1d2a	SA	Zambia	Lozi	[9]
KJ185898	L2a1d2a	SA	Zambia	Kwangwa	[9]
KJ185586	L2a1d2a	SA	Zambia	Lozi	[9]
KJ185955	L2a1d2a	SA	Zambia	Nyengo	[9]
KJ185939	L2a1d2a	SA	Zambia	Mbowe	[9]
KJ185901	L2a1d2a	SA	Zambia	Kwangwa	[9]
MOZ342	L2a1d2a	SA	Mozambique	-	Present study
Howell389	L2a1e	AM	USA	-	[4]
DQ304945	L2a1e	AM	USA	-	[7]
Howell172	L2a1e1	AM	USA	-	[4]
JQ705455	L2a1e1	-	unknown	-	[8]
JQ044821	L2a1e1	WA	Burkina Faso	-	[3]
JQ044970	L2a1e1	WA	Burkina Faso	-	[3]
DQ304946	L2a1e1	AM	USA	-	[7]
DQ304947	L2a1e1	AM	USA	-	[7]
Howell421	L2a1e1	AM	USA	-	[4]
DQ304930	L2a1e1	AM	USA	-	[7]
DQ304929	L2a1e1	AM	USA	-	[7]
DQ304931	L2a1e1	AM	USA	-	[7]
JQ044883	L2a1f	WA	Burkina Faso	-	[3]
DQ304935	L2a1f	AM	USA	-	[7]
Howell566	L2a1f	AM	USA	-	[4]
KJ185678	L2a1f	SA	Zambia	Mbunda	[9]
Howell223	L2a1f	AM	USA	-	[4]
DQ304936	L2a1f	AM	USA	-	[7]
JQ044912	L2a1f	WA	Burkina Faso	-	[3]
JQ045056	L2a1f	WA	Burkina Faso	-	[3]
JQ045087	L2a1f	WA	Yoruba	Yoruba	[3]
HG03452	L2a1f	WA	Sierra Leone	Mende	[2]

Supplementary Table 3. (continued)

KJ185728	L2a1f	SA	Zambia	Nkoya	9
KJ185899	L2a1f	SA	Zambia	Kwangwa	9
NA19023	L2a1f	EA	Kenya	Luhya	2
KJ185429	L2a1f	SA	Zambia	Luchazi	9
KJ185897	L2a1f	SA	Zambia	Kwangwa	9
KJ185893	L2a1f	SA	Zambia	Kwangwa	9
KJ185680	L2a1f	SA	Zambia	Mbunda	9
JX303832	L2a1f	SA	Zambia	Subiya	13
DQ304954	L2a1f	AM	USA	-	7
JQ705150	L2a1f	-	unknown	-	8
DQ304953	L2a1f	AM	USA	-	7
DQ304956	L2a1f	AM	USA	-	7
DQ304952	L2a1f	AM	USA	-	7
JQ704668	L2a1f	-	unknown	-	8
DQ304966	L2a1f	AM	USA	-	7
JQ703960	L2a1f	-	unknown	-	8
JQ044859	L2a1f	WA	Burkina Faso	-	3
Howell567	L2a1f	AM	USA	-	4
HG03159	L2a1f	CA	Nigeria	Esan	2
JQ045006	L2a1f	WA	Burkina Faso	-	3
HG03514	L2a1f	CA	Nigeria	Esan	2
JQ045061	L2a1f	WA	Burkina Faso	-	3
HG02979	L2a1f	CA	Nigeria	Esan	2
HG02885	L2a1f	WA	Gambia	-	2
HG03199	L2a1f	CA	Nigeria	Esan	2
AY195776	L2a1f	SA	S. Africa	-	19
KC622076	L2a1f	SA	Botswana	Tswana	10
KJ185681	L2a1f	SA	Zambia	Mbunda	9
KJ185683	L2a1f	SA	Zambia	Mbunda	9
KJ185985	L2a1f	SA	Zambia	Mambwe	9
Howell233	L2a1f	AM	USA	-	4
Howell565	L2a1f	AM	USA	-	4
DQ304959	L2a1f	AM	USA	-	7
DQ304962	L2a1f	AM	USA	-	7
DQ304965	L2a1f	AM	USA	-	7
JQ045090	L2a1f	WA	Yoruba	Yoruba	3
DQ304960	L2a1f1	AM	USA	-	7
DQ304963	L2a1f1	AM	USA	-	7
DQ304964	L2a1f1	AM	USA	-	7
EU092961	L2a1f1	AM	USA	-	1
HG03575	L2a1f1	WA	Sierra Leone	Mende	2
JQ701814	L2a1f1	AM	USA	-	8
DQ304961	L2a1f1	AM	USA	-	7
DQ304955	L2a1f1	AM	USA	-	7
KJ185685	L2a1f1	SA	Zambia	Mbunda	9
HG02557	L2a1f1	AM	Barbados	African Caribbean	2

Supplementary Table 3. (continued)

HG03135	L2a1f1	CA	Nigeria	Esan	[2]
KJ186002	L2a1f1	SA	Zambia	Tumbuka	[9]
KJ185933	L2a1f1	SA	Zambia	Makoma	[9]
DQ304967	L2a1f1	AM	USA	-	[7]
HG02476	L2a1f1	AM	Barbados	African Caribbean	[2]
HG03297	L2a1f1	CA	Nigeria	Esan	[2]
DQ304937	L2a1f1	AM	USA	-	[7]
HG03163	L2a1f1	CA	Nigeria	Esan	[2]
Howell208	L2a1f1	AM	USA	-	[4]
JX303780	L2a1f1	SA	Zambia	Tonga	[13]
DQ304958	L2a1f1a	AM	USA	-	[7]
DQ304934	L2a1f1a	AM	USA	-	[7]
JQ045097	L2a1f2	WA	Yoruba	Yoruba	[3]
JQ045039	L2a1f2	WA	Burkina Faso	-	[3]
DQ304957	L2a1f2	AM	USA	-	[7]
HG02481	L2a1f2	AM	Barbados	African Caribbean	[2]
EU597561	L2a1f3	EA	Kenya	-	[6]
JX303805	L2a1f3	SA	Zambia	Kwamashi	[13]
KJ185583	L2a1f3	SA	Zambia	Lozi	[9]
KJ185596	L2a1f3	SA	Zambia	Lozi	[9]
KJ185582	L2a1f3	SA	Zambia	Lozi	[9]
KC622118	L2a1f3	SA	Botswana	Kgalagadi	[10]
KJ185895	L2a1f3	SA	Zambia	Kwangwa	[9]
JQ044861	L2a1f3	WA	Burkina Faso	-	[3]
JX303752	L2a1g	SA	Zambia	Tonga	[13]
JX303906	L2a1g	SA	Zambia	Subiya	[13]
KJ185894	L2a1g	SA	Zambia	Kwangwa	[9]
KJ185693	L2a1g	SA	Zambia	Mbunda	[9]
JX303798	L2a1g	SA	Zambia	Totela	[13]
EU092676	L2a1h	AP / NE	Israel	Palestinian	[1]
EU092914	L2a1h	EA	Kenya	-	[1]
KJ186003	L2a1h	SA	Zambia	Tumbuka	[9]
MOZ301	L2a1h	SA	Mozambique	-	Present study
JQ044905	L2a1i	WA	Burkina Faso	-	[3]
JQ045077	L2a1i	WA	Burkina Faso	-	[3]
Howell388	L2a1i	AM	USA	-	[4]
HG03432	L2a1i	WA	Sierra Leone	Mende	[2]
HG03461	L2a1i	WA	Sierra Leone	Mende	[2]
EU092719	L2a1i	WA	G. Bissau	FulaForro	Behar, 2008
HG03064	L2a1i	WA	Sierra Leone	Mende	[2]
JQ044958	L2a1i	WA	Burkina Faso	-	[3]
JQ045080	L2a1i	WA	Burkina Faso	-	[3]
JQ045102	L2a1i	WA	Yoruba	Yoruba	[3]
JQ044881	L2a1i1	WA	Burkina Faso	-	[3]
Howell193	L2a1i1	AM	USA	-	[4]
HG02554	L2a1i1	AM	Barbados	African Caribbean	[2]

Supplementary Table 3. (continued)

AF346976	L2a1i1	-	unknown	Effik	[12]
NA18878	L2a1i1	CA	Nigeria	Yoruba	[2]
JX303857	L2a1i1	SA	Zambia	Fwe	[13]
KC622169	L2a1i1	SA	Namibia	Mbukushu	[10]
KJ185597	L2a1i1	SA	Zambia	Lozi	[9]
KJ185598	L2a1i1	SA	Zambia	Lozi	[9]
JX303909	L2a1i1	SA	Zambia	Subiya	[13]
KJ185690	L2a1i1	SA	Zambia	Mbunda	[9]
JX303853	L2a1i1	SA	Zambia	Fwe	[13]
KJ185900	L2a1i1	SA	Zambia	Kwangwa	[9]
SOM64	L2a1j	EA	Somalia	-	Present study
EU092756	L2a1j	AP / NE	Jordan	-	[1]
EU092816	L2a1j	NA	Morocco	Arab	[1]
EU200760	L2a1k	EUR	Czech Rep	-	[17]
EU200763	L2a1k	EUR	Slovenia	-	[17]
JQ045047	L2a1l	WA	Burkina Faso	-	[3]
JQ044956	L2a1l1	WA	Burkina Faso	-	[3]
JQ044919	L2a1l1	WA	Burkina Faso	-	[3]
JQ044955	L2a1l1	WA	Burkina Faso	-	[3]
HG01286	L2a1l1	AM	Puerto Rico	-	[2]
HG03428	L2a1l1	WA	Sierra Leone	Mende	[2]
HG03457	L2a1l1	WA	Sierra Leone	Mende	[2]
HG03382	L2a1l1	WA	Sierra Leone	Mende	[2]
HG03097	L2a1l1	WA	Sierra Leone	Mende	[2]
HG02568	L2a1l1	WA	Gambia	-	[2]
HG03401	L2a1l1	WA	Sierra Leone	Mende	[2]
HG03085	L2a1l1	WA	Sierra Leone	Mende	[2]
HG03547	L2a1l1	WA	Sierra Leone	Mende	[2]
JQ044817	L2a1l1a	WA	Burkina Faso	-	[3]
EU092812	L2a1l1a	NA	Morocco	Arab	[1]
FJ769771	L2a1l1a	AM	Bahamas	-	Family Tree
JQ044966	L2a1l1a	WA	Burkina Faso	-	[3]
JQ044897	L2a1l1a	WA	Burkina Faso	-	[3]
DQ304939	L2a1l1a	AM	USA	-	[7]
JQ044932	L2a1l1a	WA	Burkina Faso	-	[3]
EU092807	L2a1l1a	NA	Morocco	-	[1]
EU092721	L2a1l2	WA	G. Bissau	Mandinga	[1]
HG02716	L2a1l2	WA	Gambia	-	[2]
HG02620	L2a1l2	WA	Gambia	-	[2]
HG02629	L2a1l2	WA	Gambia	-	[2]
JQ044978	L2a1l2	WA	Burkina Faso	-	[3]
JQ044994	L2a1l2	WA	Burkina Faso	-	[3]
JQ705185	L2a1l2a	EUR	Russia	Jew	[8]
JQ705049	L2a1l2a	EUR	Poland	Jew	[8]
Howell401	L2a1l2a	AM	USA	-	[4]
Howell564	L2a1l2a	AM	USA	-	[4]

Supplementary Table 3. (continued)

EU092687	L2a1l2a	AP / NE	Israel	Ashkenazi	[1]
EU547188	L2a1l2a	EUR	Poland	Jew	Family Tree
JN204423	L2a1l2a	EUR	Poland	Jew	Family Tree
JX266264	L2a1l2a	EUR	Poland	Podhale	[20]
JQ705589	L2a1l2a1	EUR	Romania	-	[8]
EU564850	L2a1l2a2	-	unknown	-	Family Tree
JQ702015	L2a1l2a3	EUR	Poland	Jew	[8]
JQ702904	L2a1l2a4	-	unknown	-	[8]
JX266265	L2a1l2a5	EUR	Poland	Kashubia	[20]
JQ044804	L2a1m	WA	Burkina Faso	-	[3]
JQ045040	L2a1m	WA	Burkina Faso	-	[3]
JQ044916	L2a1m	WA	Burkina Faso	-	[3]
DQ304940	L2a1m1	AM	USA	-	[7]
Howell563	L2a1m1	AM	USA	-	[4]
JQ701914	L2a1m1	EUR	Ireland	-	[8]
DQ304938	L2a1m1	AM	USA	-	[7]
JQ044908	L2a1m1	WA	Burkina Faso	-	[3]
JQ703065	L2a1n	-	unknown	-	[8]
JQ044944	L2a1n	WA	Burkina Faso	-	[3]
DQ304941	L2a1n	AM	USA	-	[7]
JQ045000	L2a1o	WA	Burkina Faso	-	[3]
EU092739	L2a1o	AP / NE	Syria	-	[1]
JX303870	L2a1q	SA	Zambia	Fwe	[13]
KC622159	L2a1q	SA	Namibia	Kwanyama	[10]
NA19381	L2a1q	EA	Kenya	Luhya	[2]
SOM49	L2a1r	EA	Somalia	-	Present study
SOM17	L2a1r	EA	Somalia	-	Present study
SOM59	L2a1r	EA	Somalia	-	Present study
SOM96	L2a1r	EA	Somalia	-	Present study
SOM81	L2a1r	EA	Somalia	-	Present study
EU092896	L2a2a	CA	Chad	Sara	[1]
SUD102	L2a2a	EA	Sudan	-	Present study
HM771205	L2a2a1	CA	Pygmy	-	[14]
SUD75	L2a2a1	EA	Sudan	-	Present study
EU092902	L2a2a1	CA	Chad	Sara	[1]
EU092882	L2a2a1	CA	Chad	Sara	[1]
HM771191	L2a2b	CA	Pygmy	-	[14]
HM771207	L2a2b	CA	Pygmy	-	[14]
KJ185986	L2a2b	SA	Zambia	Mambwe	[9]
EU597525	L2a2b1	CA	Mbuti Pygmy	-	[6]
HM771193	L2a2b1	CA	Pygmy	-	[14]
AY195788	L2a2b1	SA	S. Africa	San	[19]
HM771192	L2a2b1	CA	Pygmy	-	[14]
EU597549	L2a2b1	CA	Mbuti Pygmy	-	[6]
HM771194	L2a2b1	CA	Pygmy	-	[14]
HM771208	L2a2b1	CA	Pygmy	-	[14]

Supplementary Table 3. (continued)

HM771195	L2a2b1	CA	Pygmy	-	14
HM771206	L2a3	CA	Pygmy	-	14
HM771197	L2a4a	CA	Pygmy	-	14
HM771213	L2a4a	CA	Pygmy	-	14
HM771210	L2a4a	CA	Pygmy	-	14
HM771209	L2a4a	CA	Pygmy	-	14
HM771214	L2a4a	CA	Pygmy	-	14
HM771211	L2a4a	CA	Pygmy	-	14
HM771196	L2a4a	CA	Pygmy	-	14
HM771212	L2a4a	CA	Pygmy	-	14
HM771215	L2a4a	CA	Pygmy	-	14
KJ185525	L2a5	SA	Angola	Kuvale	9
NA19045	L2a5a	EA	Kenya	Luhya	2
HQ384199	L2a5a	EUR	Spain	-	21
HM596745	L2a5a1a1	AM	Bermuda	-	Family Tree
JX303829	L2a5a1a1	SA	Zambia	Kwamashi	13
KJ185684	L2a5a1a1	SA	Zambia	Mbunda	9
KJ185687	L2a5a1a1	SA	Zambia	Mbunda	9
KJ185682	L2a5a1a1	SA	Zambia	Mbunda	9
KJ185952	L2a5a1a1	SA	Zambia	Nyengo	9
KJ185734	L2a5a1a1	SA	Zambia	Nkoya	9
KJ185953	L2a5a1a2	SA	Zambia	Nyengo	9
KJ185592	L2a5a1a2	SA	Zambia	Lozi	9
KJ185954	L2a5a1a2	SA	Zambia	Nyengo	9
KJ185942	L2a5a1a2	SA	Zambia	Mwenyi	9
KJ185932	L2a5a1a2	SA	Zambia	Makoma	9
KJ185730	L2a5a1a2	SA	Zambia	Nkoya	9
KJ185729	L2a5a1a2	SA	Zambia	Nkoya	9
KJ185427	L2a5a1b	SA	Zambia	Luchazi	9
KJ185830	L2a5a1b	SA	Angola	Ovimbundu	9
KJ185441	L2a5a1b	SA	Zambia	Luvale	9
KJ185593	L2a5a1b	SA	Zambia	Lozi	9
KJ185594	L2a5a1b	SA	Zambia	Lozi	9
KJ185823	L2a5a1b	SA	Angola	Ovimbundu	9
JN214443	L2b	EUR	Spain	Galicia	11
EU092734	L2b	WA	G. Bissau	Mandinga	1
HG02645	L2b	WA	Gambia	-	2
JQ045008	L2b	WA	Burkina Faso	-	3
EU092747	L2b1	AP / NE	S. Arabia	-	1
EU092766	L2b1	NA	Egypt	-	1
HG02982	L2b1a	WA	Gambia	-	2
JQ044854	L2b1a	WA	Burkina Faso	-	3
HG03291	L2b1a	CA	Nigeria	Esan	2
Tor66(#26)	L2b1a	AM	Dominica	-	16
HG01403	L2b1a	AM	Puerto Rico	-	2
DQ304981	L2b1a	AM	USA	-	7

Supplementary Table 3. (continued)

HG02760	L2b1a	WA	Gambia	-	[2]
Howell175	L2b1a	AM	USA	-	[4]
HG03472	L2b1a	WA	Sierra Leone	Mende	[2]
EU092664	L2b1a2	AP / NE	Israel	Bedouin	[1]
EU092722	L2b1a2	WA	G. Bissau	Manjaco	[1]
DQ304985	L2b1a2	AM	USA	-	[7]
HG02887	L2b1a2	WA	Gambia	-	[2]
HG03046	L2b1a2	WA	Gambia	-	[2]
JQ045013	L2b1a2	WA	Burkina Faso	-	[3]
JQ044890	L2b1a2	WA	Burkina Faso	-	[3]
JQ045037	L2b1a2	WA	Burkina Faso	-	[3]
DQ304978	L2b1a3	AM	USA	-	[7]
DQ304979	L2b1a3	AM	USA	-	[7]
DQ304980	L2b1a3	AM	USA	-	[7]
DQ304982	L2b1a3	AM	USA	-	[7]
DQ304983	L2b1a3	AM	USA	-	[7]
DQ304984	L2b1a3	AM	USA	-	[7]
HG03515	L2b1a3	CA	Nigeria	Esan	[2]
AY195766	L2b1a3	SA	S. Africa	-	[19]
EU092854	L2b1a3	SA	S. Africa	San	[1]
KJ185460	L2b1a3	SA	Zambia	Tokaleya	[9]
KJ185857	L2b1a3	SA	Zambia	Ovimbundu	[9]
FJ460535	L2b1a3	NA	Tunisia	-	[5]
KJ185956	L2b1a3	SA	Zambia	Nyengo	[9]
KJ185832	L2b1a3	SA	Angola	Ovimbundu	[9]
KJ185489	L2b1a3	SA	Angola	Ganguela	[9]
KJ185772	L2b1a3	SA	Angola	Nyaneka	[9]
KJ185443	L2b1a3	SA	Zambia	Luvale	[9]
KJ185600	L2b1a3	SA	Zambia	Lozi	[9]
JQ701833	L2b1a3	EUR	unknown	African European	[8]
JQ702694	L2b1a3	-	unknown	-	[8]
JX303882	L2b1a3	SA	Zambia	Shanjo	[13]
KJ185869	L2b1a3	SA	Zambia	Kwamulonga	[9]
KJ185599	L2b1a3	SA	Zambia	Lozi	[9]
NA19024	L2b1a3	EA	Kenya	Luhya	[2]
Howell568	L2b1a4	AM	USA	-	[4]
JN214453	L2b1a4	EUR	Italy	Liguria	[11]
JN214454	L2b1a4	EUR	Italy	Liguria	[11]
JQ044800	L2b1b	WA	Burkina Faso	-	[3]
HG02952	L2b1b	CA	Nigeria	Esan	[2]
Howell574	L2b1b	AM	USA	-	[4]
FJ228403	L2b1b	WA	Senegal	-	Family Tree
HM771226	L2b1b	CA	Pygmy	-	[14]
KJ185444	L2b1b	SA	Zambia	Luvale	[9]
JQ044910	L2b2	WA	Burkina Faso	-	[3]
EU092692	L2b2	SA	Mozambique	Ronga	[1]

Supplementary Table 3. (continued)

KJ185974	L2b2	SA	Zambia	Kaonde	[9]
SUD87	L2b2	EA	Sudan	-	Present study
JQ044797	L2b2	WA	Burkina Faso	-	[3]
JQ045043	L2b2	WA	Burkina Faso	-	[3]
JQ044846	L2b2a	WA	Burkina Faso	-	[3]
STP84	L2b2a	WA	STP	-	Present study
JX303841	L2b2a	SA	Zambia	Kwamashi	[13]
KJ185694	L2b2a	SA	Zambia	Mbunda	[9]
JX303807	L2b2a	SA	Zambia	Kwamashi	[13]
KJ185833	L2b2a	SA	Angola	Ovimbundu	[9]
KJ185831	L2b2a	SA	Angola	Ovimbundu	[9]
EU092661	L2b3	EA	Ethiopia	-	[1]
FJ460526	L2b3	NA	Tunisia	-	[5]
HG02804	L2b3	WA	Gambia	-	[2]
HG02595	L2b3	WA	Gambia	-	[2]
HG02837	L2b3	WA	Gambia	-	[2]
HG02555	L2b3	AM	Barbados	African Caribbean	[2]
JQ702123	L2b3a	AM	Hawaii	-	[8]
Howell222	L2b3a	AM	USA	-	[4]
JQ702626	L2b3a	-	unknown	-	[8]
Howell385	L2b3a	AM	USA	-	[4]
JQ044882	L2c	WA	Burkina Faso	-	[3]
STP43	L2c	WA	STP	-	Present study
JQ701954	L2c	-	unknown	-	[8]
HG03484	L2c	WA	Sierra Leone	Mende	[2]
EU092723	L2c	WA	G. Bissau	Fula	[1]
JQ705120	L2c	-	unknown	-	[8]
HG03376	L2c	WA	Sierra Leone	Mende	[2]
HG03451	L2c	WA	Sierra Leone	Mende	[2]
HG03473	L2c	WA	Sierra Leone	Mende	[2]
HG02703	L2c	WA	Gambia	-	[2]
JQ702169	L2c	-	unknown	-	[8]
JQ044878	L2c	WA	Burkina Faso	-	[3]
JQ044941	L2c	WA	Burkina Faso	-	[3]
HG03049	L2c	WA	Gambia	-	[2]
KJ185773	L2c	SA	Angola	Nyaneka	[9]
HG03433	L2c	WA	Sierra Leone	Mende	[2]
HG03455	L2c	WA	Sierra Leone	Mende	[2]
HG03476	L2c	WA	Sierra Leone	Mende	[2]
HG03091	L2c	WA	Sierra Leone	Mende	[2]
HG03225	L2c	WA	Sierra Leone	Mende	[2]
HG02814	L2c	WA	Gambia	-	[2]
HG02882	L2c	WA	Gambia	-	[2]
HG02888	L2c	WA	Gambia	-	[2]
HG02624	L2c	WA	Gambia	-	[2]
HG02642	L2c	WA	Gambia	-	[2]

Supplementary Table 3. (continued)

JQ044989	L2c1	WA	Burkina Faso	-	3
JQ044858	L2c1	WA	Burkina Faso	-	3
JQ045002	L2c1	WA	Burkina Faso	-	3
HG02759	L2c1	WA	Gambia	-	2
HG02505	L2c1	AM	Barbados	African Caribbean	2
EU092813	L2c1a	NA	Morocco	-	1
JN214433	L2c1a	EUR	Spain	Andalusia	11
HG03048	L2c1a	WA	Gambia	-	2
JQ045106	L2c1a	WA	Mandenka	Mandenka	3
JQ045105	L2c1a	WA	Mandenka	Mandenka	3
JQ045068	L2c1a	WA	Burkina Faso	-	3
JQ045022	L2c1a	WA	Burkina Faso	-	3
JQ044887	L2c1a	WA	Burkina Faso	-	3
JQ044901	L2c1a	WA	Burkina Faso	-	3
AF381981	L2c1a	NA	Mauritania	-	22
EU092754	L2c2	AP / NE	Lebanon	-	1
EU092697	L2c2	SA	Mozambique	Mozambique	1
JQ045042	L2c2	WA	Burkina Faso	-	3
Tor67(#08)	L2c2	AM	Dominica	-	16
DQ304986	L2c2	AM	USA	-	7
Howell573	L2c2	AM	USA	-	4
HG03069	L2c2	WA	Sierra Leone	Mende	2
HG03388	L2c2	WA	Sierra Leone	Mende	2
DQ304989	L2c2	AM	USA	-	7
JQ044920	L2c2	WA	Burkina Faso	-	3
JQ045010	L2c2	WA	Burkina Faso	-	3
HG02836	L2c2	WA	Gambia	-	2
DQ304988	L2c2a	AM	USA	-	7
EU092955	L2c2a	AM	USA	-	1
EU092957	L2c2a	AM	USA	-	1
JQ704740	L2c2a1	-	unknown	-	8
HG03157	L2c2a1	CA	Nigeria	Esan	2
DQ304987	L2c2a1	AM	USA	-	7
KJ185994	L2c2a1	SA	Zambia	Ndundulu	9
KJ185603	L2c2a1	SA	Zambia	Lozi	9
KJ185877	L2c2a1	SA	Zambia	Kwandi	9
KJ185601	L2c2a1	SA	Zambia	Lozi	9
KJ185605	L2c2a1	SA	Zambia	Lozi	9
KJ185539	L2c2a1	SA	Zambia	Kololo	9
KJ185983	L2c2a1	SA	Zambia	Lunda	9
JX303792	L2c2a1	SA	Zambia	Totela	13
KJ185604	L2c2a1	SA	Zambia	Lozi	9
JX303863	L2c2a1	SA	Zambia	Fwe	13
JX303750	L2c2a1	SA	Zambia	Totela	13
HG03354	L2c2b1	CA	Nigeria	Esan	2
JQ704094	L2c2b1a	-	unknown	-	8

Supplementary Table 3. (continued)

NA18517	L2c2b1a	CA	Nigeria	Yoruba	[2]
EU092710	L2c2b1b	EUR	Netherlands	Dutch	[1]
KJ185834	L2c2b1b	SA	Angola	Ovimbundu	[9]
KC622075	L2c2b1b	SA	Botswana	Kalanga	[10]
KJ185775	L2c2b1b	SA	Angola	Nyaneka	[9]
KJ185526	L2c2b1b	SA	Angola	Kuvale	[9]
KJ185774	L2c2b1b	SA	Angola	Nyaneka	[9]
KJ185602	L2c2b1b	SA	Angola	Lozi	[9]
JQ044853	L2c2b2	WA	Burkina Faso	-	[3]
JQ044917	L2c3	WA	Burkina Faso	-	[3]
HG03074	L2c3	WA	Sierra Leone	Mende	[2]
JQ044810	L2c3	WA	Burkina Faso	-	[3]
JQ044971	L2c3	WA	Burkina Faso	-	[3]
JQ705626	L2c3	-	unknown	-	[8]
Howell575	L2c3	AM	USA	-	[4]
Howell572	L2c3	AM	USA	-	[4]
AF346995	L2c3a	-	unknown	Mandenka	[12]
JQ045104	L2c3a	WA	Mandenka	Mandenka	[3]
JQ045109	L2c3a	WA	Mandenka	Mandenka	[3]
JQ702115	L2c4	-	unknown	-	[8]
HG02757	L2c4	WA	Gambia	-	[2]
HG02878	L2c4	WA	Gambia	-	[2]
HG02756	L2c4	WA	Gambia	-	[2]
JQ044914	L2c4	WA	Burkina Faso	-	[3]
JQ044921	L2c4	WA	Burkina Faso	-	[3]
HG02715	L2c5	WA	Gambia	-	[2]
AY195785	L2c5	SA	S. Africa	S. Africa	[19]
HG02643	L2c5	WA	Gambia	-	[2]
HG02896	L2c5	WA	Gambia	-	[2]
HG02462	L2c5	WA	Gambia	-	[2]
HG02667	L2c5	WA	Gambia	-	[2]
JQ045030	L2c6	WA	Burkina Faso	-	[3]
HG03072	L2c6	WA	Sierra Leone	Mende	[2]
HG02879	L2c6	WA	Gambia	-	[2]
STP48	L2c6	WA	STP	-	Present study
EU597570	L2d	AM	unknown	Latin America	[6]
SUD86	L2d	EA	Sudan	-	Present study
HG02536	L2d	AM	Barbados	African Caribbean	[2]
HG02881	L2d	WA	Gambia	-	[2]
EU092817	L2d	NA	Algeria	Arab	[1]
HG02799	L2d	WA	Gambia	-	[2]
HG02545	L2d1	AM	Barbados	African Caribbean	[2]
HG03437	L2d1	WA	Sierra Leone	Mende	[2]
HG03279	L2d1	CA	Nigeria	Esan	[2]
JQ045050	L2d1a	WA	Burkina Faso	-	[3]
HG02970	L2d1a	CA	Nigeria	Esan	[2]

Supplementary Table 3. (continued)

KJ185421	L2d1a	SA	Zambia	Chokwe	9
JQ044948	L2d1a	WA	Burkina Faso	-	3
JQ045044	L2d1a	WA	Burkina Faso	-	3
EU092794	L2d1a	AP / NE	Yemen	-	1
JQ045060	L2d1a	WA	Burkina Faso	-	3
Howell160	L2d1a	AM	USA	-	4
DQ341062	L2d1a	EA	Ethiopia	-	23
JQ044929	L2d1a	WA	Burkina Faso	-	3
JQ045011	L2d1a	WA	Burkina Faso	-	3
JX303748	L2d1a	SA	Zambia	Totela	13
FJ460523	L2e1a	NA	Tunisia	-	5
HG03367	L2e1a	CA	Nigeria	Esan	2
HG03130	L2e1a1	CA	Nigeria	Esan	2
KJ185902	L2e1a1a	SA	Zambia	Kwangwa	9
KJ185607	L2e1a1a	SA	Zambia	Lozi	9
KJ185695	L2e1a1a	SA	Zambia	Mbunda	9
KJ185608	L2e1a1a	SA	Zambia	Lozi	9
KJ185606	L2e1a1a	SA	Zambia	Lozi	9
SUD103	L2e1b	EA	Sudan	-	Present study
JQ044816	L2e1b	WA	Burkina Faso	-	3
Howell153	L2e1b	AM	USA	-	4
STP12	L2e1b	WA	STP	-	Present study
Tor64(#28)	L2e2	AM	Dominica	-	16
HG03209	L2e2	WA	Sierra Leone	Mende	2
HG03240	L2e3	WA	Gambia	-	2
EU092724	L2e3	WA	G. Bissau	Mandinga	1

References

1. Behar, D. M. *et al.* The Dawn of Human Matrilineal Diversity. *The American Journal of Human Genetics* **82,** 1130–1140 (2008).

2. Abecasis, G. R. *et al.* An integrated map of genetic variation from 1,092 human genomes. *Nature* **491,** 56–65 (2012).

3. Barbieri, C. *et al.* Contrasting maternal and paternal histories in the linguistic context of Burkina Faso. *Molecular Biology and Evolution* **29,** 1213–1223 (2012).

4. Howell, N., Elson, J. L., Turnbull, D. M. & Herrnstadt, C. African Haplogroup L mtDNA sequences show violations of clock-lfike evolution. *Molecular Biology and Evolution* **21,** 1843–1854 (2004).

5. Costa, M. D. *et al.* Data from complete mtDNA sequencing of Tunisian centenarians: testing haplogroup association and the "golden mean" to longevity. *Mechanisms of Ageing and Development* **130,** 222–226 (2009).

6. Hartmann, A. *et al.* Validation of microarray-based resequencing of 93 worldwide mitochondrial genomes. *Human Mutation* **30,** 115–122 (2009).

7. Just, R. S., Diegoli, T. M., Saunier, J. L., Irwin, J. A. & Parsons, T. J. Complete mitochondrial genome sequences for 265 African American and U.S. “Hispanic” individuals. *Forensic Science International: Genetics* **2,** e45–e48 (2008).

8. Behar, D. M. *et al.* A “Copernican” reassessment of the human mitochondrial DNA tree from its root. *The American Journal of Human Genetics* **90,** 675–684 (2012).

9. Barbieri, C. *et al.* Migration and Interaction in a Contact Zone: mtDNA Variation among Bantu-Speakers in Southern Africa. *PloS one* **9,** e99117 (2014).

10. Barbieri, C. *et al.* Unraveling the complex maternal history of Southern African Khoisan populations. *American Journal of Physical Anthropology* **153,** 435–448 (2014).

11. Cerezo, M. *et al.* Reconstructing ancient mitochondrial DNA links between Africa and Europe. *Genome Research* **22,** 821–826 (2012).

12. Ingman, M., Kaessmann, H., Pääbo, S. & Gyllensten, U. Mitochondrial genome variation and the origin of modern humans. *Nature* **408,** 708–713 (2000).

13. Barbieri, C., Butthof, A., Bostoen, K. & Pakendorf, B. Genetic perspectives on the origin of clicks in Bantu languages from southwestern Zambia. *European Journal of Human Genetics* **21,** 430–436 (2013).

14. Batini, C. *et al.* Insights into the demographic history of African Pygmies from complete mitochondrial genomes. *Molecular Biology and Evolution* **28,** 1099–1110 (2011).

15. Pereira, L. *et al.* No Evidence for an mtDNA Role in Sperm Motility: Data from Complete Sequencing of Asthenozoospermic Males. *Molecular Biology and Evolution* **24 ,** 868–874 (2007).

16. Torroni, A. *et al.* Do the Four Clades of the mtDNA Haplogroup L2 Evolve at Different Rates? *The American Journal of Human Genetics* **69,** 1348–1356 (2001).

17. Malyarchuk, B. A. *et al.* Reconstructing the phylogeny of African mitochondrial DNA lineages in Slavs. *European Journal of Human Genetics* **16,** 1091–1096 (2008).

18. Kujanová, M., Pereira, L., Fernandes, V., Pereira, J. B. & Cerný, V. Near eastern neolithic genetic input in a small oasis of the Egyptian Western Desert. *American Journal of Physical Anthropology* **140,** 336–346 (2009).

19. Mishmar, D. *et al.* Natural selection shaped regional mtDNA variation in humans. *Proceedings of the National Academy of Sciences of the United States of America* **100,** 171–176 (2003).

20. Mielnik-Sikorska, M., Daca, P. & Malyarchuk, B. The History of Slavs Inferred from Complete Mitochondrial Genome Sequences. *PloS one* **8,** e54360 (2013).

21. Gómez-Carballa, A. *et al.* Evolutionary analyses of entire genomes do not support the association of mtDNA mutations with Ras/MAPK pathway syndromes. *PloS one* **6,** e18348 (2011).

22. Maca-Meyer, N., Gonzalez, A., Larruga, J., Flores, C. & Cabrera, V. Major genomic mitochondrial lineages delineate early human expansions. *BMC Genetics* **2,** 13 (2001).

23. Torroni, A., Achilli, A., Macaulay, V., Richards, M. & Bandelt, H.-J. Harvesting the fruit of the human mtDNA tree. *Trends in Genetics* **22,** 339–345 (2006).

Supplementary Table 4. List of 303 complete L0a sequences used for phylogenetic reconstruction and phylogeography analysis. Codes for regions: AM – America, CA – Central Africa, EA – Eastern Africa, NA – Northern Africa, NE/AP – Near East/Arabian Peninsula, SA – Southern Africa, WA – Western Africa, EUR – Europe. Abbreviations: CAR – Central African Republic, S. Africa – South Africa, STP – São Tomé and Príncipe, S. Arabia – Saudi Arabia, USA – United States of America. Additional information on ethnicity or geographical location indicated whenever provided by the authors.

Sample	Haplogroup	Region	Country / Ethnic group	Additional information	Reference
NA19039	L0a+95	EA	Kenya	Luhya	1
JX303766	L0a1	SA	Zambia	Shanjo	2
KF672808	L0a1	SA	Mozambique	-	3
KJ186009	L0a1	SA	Angola	Ovimbundu	4
KJ185432	L0a1+16293	SA	Zambia	Luvale	4
EU092665	L0a1a	AP / NE	Israel	Bedouin	5
EU092764	L0a1a	NA	Egypt	-	5
KF672829	L0a1a	CA	Chad	Kanembou	3
EU092763	L0a1a+200	NA	Egypt	-	5
EU092881	L0a1a+200	CA	Chad	Laal	5
EU092892	L0a1a+200	CA	Chad	Sara	5
HG03063	L0a1a+200	WA	Sierra Leone	Mende	1
JQ044851	L0a1a+200	WA	Burkina Faso	-	6
JQ044903	L0a1a+200	WA	Burkina Faso	-	6
JQ702428	L0a1a+200	EUR	Italy	-	7
KF672821	L0a1a+200	EA	Ethiopia	-	3
KF672830	L0a1a+200	EA	Ethiopia	Oromo	3
NA19027	L0a1a+200	EA	Kenya	Luhya	1
NA19042	L0a1a+200	EA	Kenya	Luhya	1
NA19156	L0a1a+200	CA	Nigeria	Yoruba	1
NA19311	L0a1a+200	EA	Kenya	Luhya	1
NA19350	L0a1a+200	EA	Kenya	Luhya	1
NA19379	L0a1a+200	EA	Kenya	Luhya	1
EU092714	L0a1a1	WA	G. Bissau	Beafada	5
JQ044893	L0a1a1	WA	Burkina Faso	-	6
KF672836	L0a1a1	EA	Sudan	Arab	3
KF672837	L0a1a1	EA	Sudan	Nubian	3
AF346985	L0a1a2	-	unknown	Hausa	8
AY195780	L0a1a2	SA	S. Africa	-	9
DQ304901	L0a1a2	AM	USA	-	10
DQ304902	L0a1a2	AM	USA	-	10
DQ304903	L0a1a2	AM	USA	-	10
DQ304904	L0a1a2	AM	USA	-	10
EU092819	L0a1a2	NA	Algeria	Kabyle	5
HG01108	L0a1a2	AM	Puerto Rico	-	1
HG02971	L0a1a2	CA	Nigeria	Esan	1
HG02981	L0a1a2	CA	Nigeria	Esan	1
HG03127	L0a1a2	CA	Nigeria	Esan	1
HG03267	L0a1a2	CA	Nigeria	Esan	1
HG03268	L0a1a2	CA	Nigeria	Esan	1

Supplementary Table 4. (continued)

HG03298	L0a1a2	CA	Nigeria	Esan	1
HG03517	L0a1a2	CA	Nigeria	Esan	1
JQ044838	L0a1a2	WA	Burkina Faso	-	6
JQ044849	L0a1a2	WA	Burkina Faso	-	6
JQ044874	L0a1a2	WA	Burkina Faso	-	6
JQ044995	L0a1a2	WA	Burkina Faso	-	6
JQ045004	L0a1a2	WA	Burkina Faso	-	6
JQ045053	L0a1a2	WA	Burkina Faso	-	6
JQ702227	L0a1a2	-	unknown	-	7
JQ703481	L0a1a2	-	unknown	-	7
JX303757	L0a1a2	SA	Zambia	Subiya	2
JX303869	L0a1a2	SA	Zambia	Fwe	2
JX303911	L0a1a2	SA	Zambia	Tonga	2
KF672822	L0a1a2	WA	STP	-	3
KF672826	L0a1a2	WA	STP	-	3
KJ185430	L0a1a2	SA	Zambia	Luvale	4
KJ185656	L0a1a2	SA	Zambia	Mbunda	4
KJ185754	L0a1a2	SA	Angola	Nyaneka	4
KJ185806	L0a1a2	SA	Angola	Ovimbundu	4
KJ185863	L0a1a2	SA	Zambia	Tswana	4
KJ185884	L0a1a2	SA	Zambia	Kwangwa	4
KJ185972	L0a1a2	SA	Zambia	Kaonde	4
KJ186004	L0a1a2	SA	Zambia	Yeyi	4
KJ186005	L0a1a2	SA	Zambia	Yeyi	4
NA18861	L0a1a2	CA	Nigeria	Yoruba	1
NA18876	L0a1a2	CA	Nigeria	Yoruba	1
NA18877	L0a1a2	CA	Nigeria	Yoruba	1
NA19137	L0a1a2	CA	Nigeria	Yoruba	1
NA19216	L0a1a2	CA	Nigeria	Yoruba	1
NA19430	L0a1a2	EA	Kenya	Luhya	1
NA20334	L0a1a2	AM	USA	-	1
NA20336	L0a1a2	AM	USA	-	1
NA20355	L0a1a2	AM	USA	-	1
HG03084	L0a1a3	WA	Sierra Leone	Mende	1
Howell586	L0a1a3	AM	USA	-	11
JQ044943	L0a1a3	WA	Burkina Faso	-	6
NA18510	L0a1a3	CA	Nigeria	Yoruba	1
EU092878	L0a1b	CA	Chad	Laal	5
EU092889	L0a1b	CA	Chad	Sara	5
Howell585	L0a1b	AM	USA	-	11
AF381988	L0a1b1	NA	Morocco	-	12
DQ304900	L0a1b1	AM	USA	-	10
JX303762	L0a1b1	SA	Zambia	Fwe	2
KJ185398	L0a1b1	SA	Zambia	Bemba	4
KJ185510	L0a1b1	SA	Angola	Kuvale	4
DQ304899	L0a1b1a	AM	USA	-	10
EU935434	L0a1b1a	NA	Egypt	el-Hayez oasis	13
EU935437	L0a1b1a	NA	Egypt	el-Hayez oasis	13
EU935464	L0a1b1a	NA	Egypt	el-Hayez oasis	13

Supplementary Table 4. (continued)

EU935467	L0a1b1a	NA	Egypt	el-Hayez oasis	13
JX303817	L0a1b1a	SA	Zambia	Fwe	2
JX303823	L0a1b1a	SA	Zambia	Fwe	2
KF672807	L0a1b1a	CA	Chad	Daza	3
KF672827	L0a1b1a	CA	Cameroon	Kotoco	3
KJ185504	L0a1b1a	SA	Angola	Kuvale	4
KJ185509	L0a1b1a	SA	Angola	Kuvale	4
KJ185753	L0a1b1a	SA	Angola	Nyaneka	4
KJ185756	L0a1b1a	SA	Angola	Nyaneka	4
KJ185757	L0a1b1a	SA	Angola	Nyaneka	4
KJ185759	L0a1b1a	SA	Angola	Nyaneka	4
NA19703	L0a1b1a	AM	USA	-	1
EU092688	L0a1b1a1	SA	Mozambique	Shangaan	5
EU092858	L0a1b1a1	SA	S. Africa	San	5
EU092869	L0a1b1a1	SA	S. Africa	SWB	5
EU092871	L0a1b1a1	SA	S. Africa	SEB	5
EU092909	L0a1b1a1	EA	Kenya	-	5
Howell587	L0a1b1a1	AM	USA	-	11
JQ702326	L0a1b1a1	-	unknown	-	7
JX303796	L0a1b1a1	SA	Zambia	Kwamashi	2
KC622063	L0a1b1a1	SA	Botswana	Tswana	14
KC622067	L0a1b1a1	SA	Botswana	Tswana	14
KC622068	L0a1b1a1	SA	Botswana	Tswana	14
KC622077	L0a1b1a1	SA	Botswana	Kgalagadi	14
KC622078	L0a1b1a1	SA	Botswana	Kalanga	14
KC622112	L0a1b1a1	SA	Botswana	Kgalagadi	14
KC622114	L0a1b1a1	SA	Botswana	Kgalagadi	14
KC622115	L0a1b1a1	SA	Botswana	Kgalagadi	14
KC622117	L0a1b1a1	SA	Botswana	Kgalagadi	14
KC622121	L0a1b1a1	SA	Namibia	Owambo	14
KF672805	L0a1b1a1	SA	Mozambique	-	3
KF672806	L0a1b1a1	SA	Mozambique	-	3
KJ185423	L0a1b1a1	SA	Zambia	Luchazi	4
KJ185425	L0a1b1a1	SA	Zambia	Luchazi	4
KJ185431	L0a1b1a1	SA	Zambia	Luvale	4
KJ185540	L0a1b1a1	SA	Zambia	Lozi	4
KJ185542	L0a1b1a1	SA	Zambia	Lozi	4
KJ185545	L0a1b1a1	SA	Zambia	Lozi	4
KJ185547	L0a1b1a1	SA	Zambia	Lozi	4
KJ185548	L0a1b1a1	SA	Zambia	Lozi	4
KJ185549	L0a1b1a1	SA	Zambia	Lozi	4
KJ185552	L0a1b1a1	SA	Zambia	Lozi	4
KJ185554	L0a1b1a1	SA	Zambia	Lozi	4
KJ185555	L0a1b1a1	SA	Zambia	Lozi	4
KJ185659	L0a1b1a1	SA	Zambia	Mbunda	4
KJ185660	L0a1b1a1	SA	Zambia	Mbunda	4
KJ185718	L0a1b1a1	SA	Zambia	Nkoya	4
KJ185858	L0a1b1a1	SA	Zambia	Shanjo	4
KJ185966	L0a1b1a1	SA	Zambia	Yauma	4

Supplementary Table 4. (continued)

KJ185967	L0a1b1a1	SA	Zambia	Shona	4
KJ185977	L0a1b1a1	SA	Zambia	Lunda	4
KJ185978	L0a1b1a1	SA	Zambia	Lunda	4
KJ185979	L0a1b1a1	SA	Zambia	Lunda	4
KJ185990	L0a1b1a1	SA	Zambia	-	4
KJ186001	L0a1b1a1	SA	Zambia	Tebele	4
NA19382	L0a1b1a1	EA	Kenya	Luhya	1
NA19448	L0a1b1a1	EA	Kenya	Luhya	1
NA19449	L0a1b1a1	EA	Kenya	Luhya	1
NA19466	L0a1b1a1	EA	Kenya	Luhya	1
DQ304897	L0a1b2	AM	USA	-	10
DQ304898	L0a1b2	AM	USA	-	10
EU092963	L0a1b2	AM	USA	-	5
KJ185497	L0a1b2	SA	Angola	Kuvale	4
KJ185502	L0a1b2	SA	Angola	Kuvale	4
KJ185507	L0a1b2	SA	Angola	Kuvale	4
KJ185750	L0a1b2	SA	Angola	Nyaneka	4
KJ185758	L0a1b2	SA	Angola	Nyaneka	4
KJ186008	L0a1b2	SA	Angola	Nyaneka	4
EU092746	L0a1b2a	AP / NE	S. Arabia	-	5
Howell560	L0a1b2a	AM	USA	-	11
KJ185551	L0a1b2a	SA	Zambia	Lozi	4
KJ185805	L0a1b2a	SA	Angola	Ovimbundu	4
KJ185926	L0a1b2a	SA	Zambia	Makoma	4
EU092760	L0a1c1	AS	Iran	-	5
EU092945	L0a1c1	EA	Ethiopia	-	5
NA19467	L0a1c1	EA	Kenya	Luhya	1
KF672811	L0a1c2	CA	Chad	Daza	3
NA19440	L0a1c2	EA	Kenya	Luhya	1
EU092670	L0a1d	EA	Ethiopia	Jew	5
EU092801	L0a1d	AP / NE	Yemen	-	5
EU092809	L0a1d	AP / NE	Yemen	-	5
EU092810	L0a1d	AP / NE	Yemen	-	5
EU092950	L0a1d	EA	Ethiopia	-	5
KF672812	L0a1d	EA	Ethiopia	Oromo	3
KF672815	L0a1d	EA	Kenya	Turkana	3
KF672820	L0a1d	EA	Somalia	-	3
EF184602	L0a2	EA	Tanzania	-	15
EF184604	L0a2	EA	Tanzania	-	15
EF184606	L0a2	EA	Tanzania	-	15
EF184607	L0a2	EA	Tanzania	-	15
EF184608	L0a2	EA	Tanzania	-	15
KF672810	L0a2	EA	Somalia	-	3
HM771160	L0a2a1	CA	CAR	Pygmy	16
HM771161	L0a2a1	CA	CAR	Pygmy	16
KC622154	L0a2a1a	SA	Namibia	Owambo	14
KF672831	L0a2a1a	CA	Niger	Zinder	3
KJ185396	L0a2a1a	SA	Zambia	Bemba	4
KJ185433	L0a2a1a	SA	Zambia	Luvale	4

Supplementary Table 4. (continued)

KJ185722	L0a2a1a	SA	Zambia	Nkoya	4
KJ185751	L0a2a1a	SA	Angola	Nyaneka	4
KJ185752	L0a2a1a	SA	Angola	Nyaneka	4
KJ185881	L0a2a1a	SA	Zambia	Kwangwa	4
KJ185882	L0a2a1a	SA	Zambia	Kwangwa	4
KJ185883	L0a2a1a	SA	Zambia	Kwangwa	4
KJ185424	L0a2a1a1	SA	Zambia	Luchazi	4
EU092868	L0a2a1a2	SA	S. Africa	SWB	5
EU092911	L0a2a1a2	EA	Kenya	-	5
KC622065	L0a2a1a2	SA	Botswana	Tswana	14
KF672834	L0a2a1a2	WA	STP	-	3
KJ185968	L0a2a1a2	SA	Zambia	Shona	4
EU092861	L0a2a1b	SA	S. Africa	SWB	5
JX303784	L0a2a1b	SA	Zambia	Tonga	2
JX303830	L0a2a1b	SA	Zambia	Kwamashi	2
KJ185397	L0a2a1b	SA	Zambia	Bemba	4
KJ185461	L0a2a1b	SA	Zambia	Tonga	4
KJ185462	L0a2a1b	SA	Zambia	Tonga	4
KJ185476	L0a2a1b	SA	Angola	Ganguela	4
KJ185480	L0a2a1b	SA	Angola	Ganguela	4
KJ185494	L0a2a1b	SA	Angola	Kuvale	4
KJ185495	L0a2a1b	SA	Angola	Kuvale	4
KJ185496	L0a2a1b	SA	Angola	Kuvale	4
KJ185498	L0a2a1b	SA	Angola	Kuvale	4
KJ185499	L0a2a1b	SA	Angola	Kuvale	4
KJ185500	L0a2a1b	SA	Angola	Kuvale	4
KJ185501	L0a2a1b	SA	Angola	Kuvale	4
KJ185503	L0a2a1b	SA	Angola	Kuvale	4
KJ185505	L0a2a1b	SA	Angola	Kuvale	4
KJ185506	L0a2a1b	SA	Angola	Kuvale	4
KJ185508	L0a2a1b	SA	Angola	Kuvale	4
KJ185543	L0a2a1b	SA	Zambia	Lozi	4
KJ185546	L0a2a1b	SA	Zambia	Lozi	4
KJ185557	L0a2a1b	SA	Zambia	Tonga	4
KJ185653	L0a2a1b	SA	Zambia	Mbunda	4
KJ185654	L0a2a1b	SA	Zambia	Mbunda	4
KJ185717	L0a2a1b	SA	Zambia	Nkoya	4
KJ185720	L0a2a1b	SA	Zambia	Nkoya	4
KJ185755	L0a2a1b	SA	Angola	Nyaneka	4
KJ185865	L0a2a1b	SA	Zambia	Kwamulonga	4
FJ157838	L0a2a2	AS	India	-	17
FJ157839	L0a2a2	AS	India	-	17
FJ157840	L0a2a2	AS	India	-	17
DQ341058	L0a2a2a	AM	Dominica	-	18
EU092701	L0a2a2a	SA	Mozambique	Shangaan	5
EU092745	L0a2a2a	AP / NE	S. Arabia	-	5
EU092787	L0a2a2a	AP / NE	Oman	-	5
EU092925	L0a2a2a	AP / NE	Oman	-	5
Howell149	L0a2a2a	AM	USA	-	11

Supplementary Table 4. (continued)

JQ705109	L0a2a2a	-	unknown	-	7
JX303763	L0a2a2a	SA	Zambia	Totela	2
JX303772	L0a2a2a	SA	Zambia	Totela	2
JX303778	L0a2a2a	SA	Zambia	Totela	2
JX303786	L0a2a2a	SA	Zambia	Tonga	2
JX303826	L0a2a2a	SA	Zambia	Kwamashi	2
JX303904	L0a2a2a	SA	Zambia	Totela	2
KC622056	L0a2a2a	SA	Botswana	Tswana	14
KC622064	L0a2a2a	SA	Botswana	Tswana	14
KC622187	L0a2a2a	SA	Namibia	Kwanyama	14
KF672819	L0a2a2a	SA	Mozambique	-	3
KF672824	L0a2a2a	WA	STP	-	3
KF672825	L0a2a2a	EA	Somalia	-	3
KF672832	L0a2a2a	SA	Mozambique	-	3
KF672835	L0a2a2a	EA	Somalia	-	3
KJ185394	L0a2a2a	SA	Zambia	Aushi	4
KJ185458	L0a2a2a	SA	Zambia	Tokaleya	4
KJ185477	L0a2a2a	SA	Angola	Ganguela	4
KJ185478	L0a2a2a	SA	Angola	Ganguela	4
KJ185479	L0a2a2a	SA	Angola	Ganguela	4
KJ185544	L0a2a2a	SA	Zambia	Lozi	4
KJ185556	L0a2a2a	SA	Zambia	Lozi	4
KJ185657	L0a2a2a	SA	Zambia	Mbunda	4
KJ185658	L0a2a2a	SA	Zambia	Mbunda	4
KJ185721	L0a2a2a	SA	Zambia	Nkoya	4
KJ185749	L0a2a2a	SA	Angola	Nyaneka	4
KJ185802	L0a2a2a	SA	Angola	Ovimbundu	4
KJ185804	L0a2a2a	SA	Angola	Ovimbundu	4
KJ185862	L0a2a2a	SA	Zambia	Tswana	4
KJ185970	L0a2a2a	SA	Zambia	Chewa	4
NA19312	L0a2a2a	EA	Kenya	Luhya	1
NA19328	L0a2a2a	EA	Kenya	Luhya	1
NA19402	L0a2a2a	EA	Kenya	Luhya	1
NA19904	L0a2a2a	AM	USA	-	1
JX303831	L0a2a2a1	SA	Zambia	Kwamashi	2
JX303835	L0a2a2a1	SA	Zambia	Kwamashi	2
KJ185541	L0a2a2a1	SA	Zambia	Lozi	4
KJ185550	L0a2a2a1	SA	Zambia	Lozi	4
KJ185553	L0a2a2a1	SA	Zambia	Lozi	4
KJ185655	L0a2a2a1	SA	Zambia	Mbunda	4
KJ185719	L0a2a2a1	SA	Zambia	Nkoya	4
KJ185803	L0a2a2a1	SA	Angola	Ovimbundu	4
KJ185946	L0a2a2a1	SA	Zambia	Nyengo	4
KJ185947	L0a2a2a1	SA	Zambia	Nyengo	4
NA19713	L0a2a2a1	AM	USA	-	1
NA19985	L0a2a2a1	AM	USA	-	1
AF346998	L0a2b	CA	Congo	Mbuti Pygmy	8
AF346999	L0a2b	CA	Congo	Mbuti Pygmy	8
AM711903	L0a2b	CA	Congo	Pygmy	19

Supplementary Table 4. (continued)

EU597537	L0a2b	CA	Congo	Pygmy	[20]
HM771189	L0a2b	CA	Congo	Pygmy	[16]
HM771190	L0a2b	CA	Congo	Pygmy	[16]
HM771199	L0a2b	CA	Congo	Pygmy	[16]
HM771202	L0a2b	CA	Congo	Pygmy	[16]
HM771188	L0a2b1	CA	Congo	Pygmy	[16]
HM771200	L0a2b1	CA	Congo	Pygmy	[16]
HM771201	L0a2b1	CA	Congo	Pygmy	[16]
EF556174	L0a2c	EA	Ethiopia	Jew	[21]
KF672813	L0a2c	EA	Somalia	-	[3]
EU092913	L0a2d	EA	Kenya	-	[5]
KJ185399	L0a2d	SA	Zambia	Bemba	[4]
KJ185463	L0a2d	SA	Zambia	Tonga	[4]
EU092900	L0a3	CA	Chad	Sara	[5]
KF672796	L0a3	CA	Cameroon	Bulahay	[3]
EU092906	L0a4	EA	Kenya	-	[5]

References

1. Abecasis, G. R. *et al.* An integrated map of genetic variation from 1,092 human genomes. *Nature* **491,** 56–65 (2012).

2. Barbieri, C., Butthof, A., Bostoen, K. & Pakendorf, B. Genetic perspectives on the origin of clicks in Bantu languages from southwestern Zambia. *European Journal of Human Genetics* **21,** 430–436 (2013).

3. Rito, T. *et al.* The first modern human dispersals across Africa. *PloS one* **8,** e80031 (2013).

4. Barbieri, C. *et al.* Migration and Interaction in a Contact Zone: mtDNA Variation among Bantu-Speakers in Southern Africa. *PloS one* **9,** e99117 (2014).

5. Behar, D. M. *et al.* The Dawn of Human Matrilineal Diversity. *The American Journal of Human Genetics* **82,** 1130–1140 (2008).

6. Barbieri, C. *et al.* Contrasting maternal and paternal histories in the linguistic context of Burkina Faso. *Molecular Biology and Evolution* **29,** 1213–1223 (2012).

7. Behar, D. M. *et al.* A "Copernican" reassessment of the human mitochondrial DNA tree from its root. *The American Journal of Human Genetics* **90,** 675–684 (2012).

8. Ingman, M., Kaessmann, H., Pääbo, S. & Gyllensten, U. Mitochondrial genome variation and the origin of modern humans. *Nature* **408,** 708–713 (2000).

9. Mishmar, D. *et al.* Natural selection shaped regional mtDNA variation in humans. *Proceedings of the National Academy of Sciences of the United States of America* **100,** 171–176 (2003).

10. Just, R. S., Diegoli, T. M., Saunier, J. L., Irwin, J. A. & Parsons, T. J. Complete mitochondrial genome sequences for 265 African American and U.S. "Hispanic" individuals. *Forensic Science International: Genetics* **2,** e45–e48 (2008).

11. Howell, N., Elson, J. L., Turnbull, D. M. & Herrnstadt, C. African Haplogroup L mtDNA sequences show violations of clock-lfike evolution. *Molecular Biology and Evolution* **21,** 1843–1854 (2004).

12. Maca-Meyer, N., Gonzalez, A., Larruga, J., Flores, C. & Cabrera, V. Major genomic mitochondrial lineages delineate early human expansions. *BMC Genetics* **2,** 13 (2001).

13. Kujanová, M., Pereira, L., Fernandes, V., Pereira, J. B. & Cerný, V. Near eastern neolithic genetic input in a small oasis of the Egyptian Western Desert. *American Journal of Physical Anthropology* **140,** 336–346 (2009).

14. Barbieri, C. *et al.* Unraveling the complex maternal history of Southern African Khoisan populations. *American Journal of Physical Anthropology* **153,** 435–448 (2014).

15. Gonder, M. K., Mortensen, H. M., Reed, F. a, de Sousa, A. & Tishkoff, S. a. Whole-mtDNA genome sequence analysis of ancient African lineages. *Molecular Biology and Evolution* **24,** 757–768 (2007).

16. Batini, C. *et al.* Insights into the demographic history of African Pygmies from complete mitochondrial genomes. *Molecular Biology and Evolution* **28,** 1099–1110 (2011).

17. Eaaswarkhanth, M. *et al.* Traces of sub-Saharan and Middle Eastern lineages in Indian Muslim populations. *European Journal of Human Genetics : EJHG* **18,** 354–363 (2010).

18. Torroni, A., Achilli, A., Macaulay, V., Richards, M. & Bandelt, H.-J. Harvesting the fruit of the human mtDNA tree. *Trends in Genetics* **22,** 339–345 (2006).

19. Arnason, U., Gullberg, A., Janke, A. & Kullberg, M. Mitogenomic analyses of caniform relationships. *Molecular Phylogenetics and Evolution* **45,** 863–874 (2007).

20. Hartmann, A. *et al.* Validation of microarray-based resequencing of 93 worldwide mitochondrial genomes. *Human Mutation* **30,** 115–122 (2009).

21. Behar, D. M. *et al.* Counting the founders: the matrilineal genetic ancestry of the Jewish Diaspora. *PloS one* **3,** e2062 (2008).

Supplementary Table 5. GPS coordinates in WGS-84 (World Geodetic System, 1984) of capital cities used as reference points to construct L2 frequency distribution and references for the 12880 HVS-I sequences used to assess L2 frequency. Codes for regions: CA – Central Africa, EA – Eastern Africa, NA – Northern Africa, SA – Southern Africa, WA – Western Africa. Abbreviations: BF – Burkina Faso, CV – Cape Verde, Eq. Guinea – Equatorial Guinea, STP – São Tomé and Príncipe, S. Leone – Sierra Leone, W. Sahara – Western Sahara.

Region	Country	Capital	GPS Coordinates (WGS 84)	References
WA	BF	Ouagadougou	12°21′26″ N 1°32′07″ W	1–3
	CV	Praia	14°55’15" N 23° 30’30" W	4
	Gambia	Banjul	13°27′11″ N 16°34′39″ W	5
	Ghana	Accra	5°33′00″ N 0°12′00″ W	6
	Mali	Bamako	12°39′00″ N 8°00′00″ W	1,3,7,8
	STP	São Tomé	0°20′10″ N 6°40′53″ E	9,10
	Senegal	Dakar	14°41′34″ N 17°26′48″ W	2,11,12
	S.Leone	Freetown	8°29′4″ N 13°14′04″ W	5,13
CA	Cameroon	Yaoundé	3°52′00‘‘ N 11°31′00‘‘ E	3,6,14
	Chad	N’Djamena	12°06′47″ N 15°02′57″ E	3,11
	Congo	Brazzaville	4°16′04″ S 15°17′31″ E	11
	Eq. Guinea	Malabo	3°45′00‘‘ N 8°46′59″ E	9
	Niger	Niamey	13°31′17″ N 02°06′19″ E	1,3,11
	Nigeria	Abuja	9°04′00″ N 7°29′00″ E	3,5,6,11
	W. Sahara	El Aaiún	27°09′13″ N 13°12′12″ W	12,15
EA	Ethiopia	Addis Ababa	9°01′48″ N 38°44′24″ E	16–19
	Kenya	Nairobi	1°17′31‘‘ S 36°49′19’’ E	5,11,20,21
	Rwanda	Kigali	1°56′38″ S 30°03′34″ E	22
	Somalia	Mogadishu	02°02′ N 45°21′ E	11,19
	Sudan	Khartoum	15°32′47‘‘ N 32°32′00’’ E	19,23
	Tanzania	Dodoma	06° 10’23" S 35° 44’31" E	24
SA	Angola	Luanda	8°50′18″ S 13°14′04″ E	25–27
	Botswana	Gaborone	24°39′29″ S 25°54′44″ E	28
	Cabinda	Cabinda	5°33′36″ S 12°11′24″ E	29
	Madagascar	Antananarivo	18°54′51‘‘ S 47°31′51‘‘ E	30
	Mozambique	Maputo	25°57′55″ S 32°35′21″ E	31,32
	Namibia	Windhoek	22°34′12″ S 17°05′01″ E	28
	South Africa	Pretoria	25°44′46″ S 28°11′17″ E	33,34
	Zambia	Lusaka	15°24′29″ S 28°17′10″ E	35,36
	Zimbabwe	Harare	17°51′50″ S 31°01′47″ E	22
NA	Algeria	Algiers	36°45′08″ N 3°02′31″ E	15
	Egypt	Cairo	30°03′29″ N 31°13′44″ E	37,38
	Libya	Tripoli	32°54′08″ N 13°11′09″ E	39
	Mauritania	Nouakchott	18°06′01″ N 15°56′59″ W	8,12
	Morocco	Rabat	34°00′47″ N 6°49′57″ W	12,37,40
	Tunisia	Tunis	36°49′08″ N 10°09′56″ E	15,41–44

References

1. Pereira, L. *et al.* Linking the sub-Saharan and West Eurasian gene pools: maternal and paternal heritage of the Tuareg nomads from the African Sahel. *European Journal of Human Genetics* **18,** 915–923 (2010).

2. Barbieri, C. *et al.* Contrasting maternal and paternal histories in the linguistic context of Burkina Faso. *Molecular Biology and Evolution* **29,** 1213–1223 (2012).

3. Černý, V. *et al.* Genetic Structure of Pastoral and Farmer Populations in the African Sahel. *Molecular Biology and Evolution* **28,** 2491–2500 (2011).

4. Brehm, A., Pereira, L., Bandelt, H. J., Prata, M. J. & Amorim, A. Mitochondrial portrait of the Cabo Verde archipelago: the Senegambian outpost of Atlantic slave trade. *Annals of Human Genetics* **66,** 49–60 (2002).

5. Abecasis, G. R. *et al.* An integrated map of genetic variation from 1,092 human genomes. *Nature* **491,** 56–65 (2012).

6. Veeramah, K. R. *et al.* Little genetic differentiation as assessed by uniparental markers in the presence of substantial language variation in peoples of the Cross River region of Nigeria. *BMC Evolutionary Biology* **10,** 92 (2010).

7. Ely, B., Wilson, J. L., Jackson, F. & Jackson, B. A. African-American mitochondrial DNAs often match mtDNAs found in multiple African ethnic groups. *BMC Biology* **4,** 34 (2006).

8. González, A. M. *et al.* Mitochondrial DNA variation in Mauritania and Mali and their genetic relationship to other Western Africa populations. *Annals of Human Genetics* **70,** 631–657 (2006).

9. Mateu, E. *et al.* A tale of two islands: population history and mitochondrial DNA sequence variation of Bioko and São Tomé, Gulf of Guinea. *Annals of Human Genetics* **61,** 507–518 (1997).

10. Trovoada, M. J. *et al.* Pattern of mtDNA Variation in Three Populations from Sao Tome e Principe. *Annals of Human Genetics* **68,** 40–54 (2004).

11. Watson, E., Forster, P., Richards, M. & Bandelt, H. Mitochondrial footprints of human expansions in Africa. The *American Journal of Human Genetics* 691–704 (1997).

12. Rando, J. C. *et al.* Mitochondrial DNA analysis of Northwest African populations reveals genetic exchanges with European, Near-Eastern, and sub-Saharan populations. *Annals of Human Genetics* **62,** 531–550 (1998).

13. Jackson, B. A. *et al.* Mitochondrial DNA genetic diversity among four ethnic groups in Sierra Leone. *American Journal of Physical Anthropology* **128,** 156–163 (2005).

14. Quintana-Murci, L. *et al.* Maternal traces of deep common ancestry and asymmetric gene flow between Pygmy hunter-gatherers and Bantu-speaking farmers. *Proceedings of the National Academy of Sciences of the United States of America* **105,** 1596–1601 (2008).

15. Plaza, S. *et al.* Joining the Pillars of Hercules: mtDNA Sequences Show Multidirectional Gene Flow in the Western Mediterranean. *Annals of Human Genetics* **67,** 312–328 (2003).

16. Poloni, E. S. *et al.* Genetic evidence for complexity in ethnic differentiation and history in East Africa. *Annals of human genetics* **73,** 582–600 (2009).

17. Kivisild, T. *et al.* Ethiopian Mitochondrial DNA Heritage: Tracking Gene Flow Across and Around the Gate of Tears. *The American Journal of Human Genetics* **75,** 752–770 (2004).

18. Non, A. L., Al-Meeri, A., Raaum, R. L., Sanchez, L. F. & Mulligan, C. J. Mitochondrial DNA reveals distinct evolutionary histories for Jewish populations in Yemen and Ethiopia. *American Journal of Physical Anthropology* **144,** 1–10 (2011).

19. Soares, P. *et al.* The Expansion of mtDNA Haplogroup L3 within and out of Africa. *Molecular Biology and Evolution* **29,** 915–927 (2012).

20. Brandstätter, A. *et al.* Mitochondrial DNA control region sequences from Nairobi (Kenya): inferring phylogenetic parameters for the establishment of a forensic database. *International Journal of Legal Medicine* **118,** 294–306 (2004).

21. Boattini, A. *et al.* mtDNA variation in East Africa unravels the history of Afro-Asiatic groups. *American Journal of Physical Anthropology* **150,** 375–385 (2013).

22. Castrì, L. *et al.* mtDNA variability in two Bantu-speaking populations (Shona and Hutu) from Eastern Africa: implications for peopling and migration patterns in sub-Saharan Africa. *American Journal of Physical Anthropology* **140,** 302–311 (2009).

23. Krings, M. *et al.* mtDNA Analysis of Nile River Valley Populations: A Genetic Corridor or a Barrier to Migration? *The American Journal of Human Genetics* **64,** 1166–1176 (1999).

24. Knight, A., Underhill, P. & Mortensen, H. African Y chromosome and mtDNA divergence provides insight into the history of click languages. *Current Biology* **13,** 464–473 (2003).

25. Coelho, M., Sequeira, F., Luiselli, D., Beleza, S. & Rocha, J. On the edge of Bantu expansions: mtDNA, Y chromosome and lactase persistence genetic variation in southwestern Angola. *BMC Evolutionary Bioloiy* **9,** 80 (2009).

26. Plaza, S. *et al.* Insights into the western Bantu dispersal: mtDNA lineage analysis in Angola. *Human Genetics* **115,** 439–447 (2004).

27. Barbieri, C. *et al.* Migration and Interaction in a Contact Zone: mtDNA Variation among Bantu-Speakers in Southern Africa. *PloS one* **9,** e99117 (2014).

28. Barbieri, C. *et al.* Unraveling the complex maternal history of Southern African Khoisan populations. *American Journal of Physical Anthropology* **153,** 435–448 (2014).

29. Beleza, S., Gusmão, L., Amorim, A., Carracedo, A. & Salas, A. The genetic legacy of western Bantu migrations. *Human Genetics* **117,** 366–375 (2005).

30. Tofanelli, S. *et al.* On the origins and admixture of Malagasy: new evidence from high-resolution analyses of paternal and maternal lineages. *Molecular Biology and Evolution* **26,** 2109–2124 (2009).

31. Salas, A. *et al.* The making of the African mtDNA landscape. *The American Journal of Human Genetics* **71,** 1082–1111 (2002).

32. Pereira, L. *et al.* Prehistoric and historic traces in the mtDNA of Mozambique : insights into the Bantu expansions and the slave trade. *Annals of Human Genetics* **65,** 439–458 (2001).

33. Quintana-Murci, L. *et al.* Strong maternal Khoisan contribution to the South African coloured population: a case of gender-biased admixture. *The American Journal of Human Genetics* **86,** 611–620 (2010).

34. Chen, Y. S. *et al.* mtDNA variation in the South African Kung and Khwe-and their genetic relationships to other African populations. *The American Journal of Human Genetics* **66,** 1362–1383 (2000).

35. De Filippo, C., Heyn, P., Barham, L., Stoneking, M. & Pakendorf, B. Genetic perspectives on forager-farmer interaction in the Luangwa valley of Zambia. *American Journal of Physical Anthropology* **141,** 382–394 (2010).

36. Barbieri, C., Butthof, A., Bostoen, K. & Pakendorf, B. Genetic perspectives on the origin of clicks in Bantu languages from southwestern Zambia. *European Journal of Human Genetics* **21,** 430–436 (2013).

37. Coudray, C. *et al.* The complex and diversified mitochondrial gene pool of Berber populations. *Annals of Human Genetics* **73,** 196–214 (2009).

38. Saunier, J. L. *et al.* Mitochondrial control region sequences from an Egyptian population sample. *Forensic Science International: Genetics* **3,** e97–e103 (2009).

39. Ottoni, C. *et al.* First genetic insight into Libyan Tuaregs: a maternal perspective. *Annals of Human Genetics* **73,** 438–448 (2009).

40. Rhouda, T. *et al.* Moroccan mitochondrial genetic background suggests prehistoric human migrations across the Gibraltar Strait. *Mitochondrion* **9,** 402–407 (2009).

41. Fadhlaoui-Zid, K. *et al.* Mitochondrial DNA heterogeneity in Tunisian Berbers. *Annals of Human Genetics* **68,** 222–233 (2004).

42. Loueslati, B. Y. *et al.* Islands inside an island: reproductive isolates on Jerba island. *American journal of human biology : the official journal of the Human Biology Council* **18,** 149–153 (2006).

43. Turchi, C. *et al.* Polymorphisms of mtDNA control region in Tunisian and Moroccan populations: an enrichment of forensic mtDNA databases with Northern Africa data. *Forensic Science International: Genetics* **3,** 166–172 (2009).

44. Cherni, L. *et al.* Post-Last Glacial Maximum Expansion From Iberia to North Africa Revealed by Fine Characterization of mtDNA H Haplogroup in Tunisia. *American Journal of Physical Anthropology* **139,** 253–260 (2009).

Supplementary Table 6. Information on the populations used to assess pairwise genetic distances and to compute the MDS plots. References for the 4880 HVS-I sequences used. Codes for regions: CA – Central Africa, EA – Eastern Africa, SA – Southern Africa, WA – Western Africa. Abbreviations: Eq. Guinea – Equatorial Guinea, STP – São Tomé and Príncipe.

Region	Country	Population	Code	N	Language group	Additional information	Reference
EA	Ethiopia	Daasanach	Daa	49	Cushitic	Agropastoralist	1
		Dawro-Konta	Daw	137	Omotic	Agropastoralist	2
		Nyangatom	Nya	112	Nilotic	Agropastoralist	1
		General population	Eth	77	-	-	3
	Kenya	El Molo	Elm	52	Cushitic	Fishing	2
		Luo	Luo	49	Nilotic	Agropastoralist, fishing	2
		Luhya	LWK	120	Bantu	Agriculturalist	4
		Maasai	Maa	81	Nilotic	Seminomadic pastoralist	2
		Turkana	Turk	37	Nilotic	Seminomadic pastoralist	5
	Rwanda	Hutu	Hutu	42	Bantu	-	6
	Somalia	General population	Som	177	-	-	3,5
	Sudan	General population	Sud	178	-	-	3,7
	Tanzania	Burunge	Buru	38	Cushitic	Agriculturalist	8
		Datog	Dat	31	Nilotic	Pastoralist, Agriculturalist	8
WA	Burkina Faso	Various groups	BF	291	-	-	9
	Cape Verde	General population	CV	295	-	-	10
	Gambia	Western Division	GWD	113	-	-	4
	Mali	Bambara	Mali	158	Mande	-	11,12
		Malinke			Mande	-	
	Senegal	Mandenka	Man	131	Mande	-	5,9
		Wolof	Wol	61	Wolof	-	13,14
	Sierra Leone	Mende	MSL	85	Mel	-	4
CA	Eq. Guinea	Bioko	Bio	45	Bantu	-	15
	Cameroon	Ngumba	Ngu	88	Bantu	-	16
	Gabon	Akele	Ake	48	Bantu	-	16
		Ateke	Ate	54	Bantu	-	
		Benga	Beng	50	Bantu	-	
		Duma	Duma	47	Bantu	-	
		Eshira	Eshi	40	Bantu	-	
		Eviya	Evi	38	Bantu	-	
		Fang	Fang	66	Bantu	-	
		Galoa	Gal	51	Bantu	-	
		Kota	Kota	56	Bantu	-	
		Makina	Mak	45	Bantu	-	
		Mitsogo	Mits	64	Bantu	-	

Table S6. (continued)

		Nzebi	Nze	63	Bantu	-	
		Punu	Punu	52	Bantu	-	
		Shake	Sha	51	Bantu	-	
	Nigeria	Esan	ESN	99	Edoid	-	[4]
		Yoruba	Yor	193	Yoruboid	-	[4,5,9]
	STP	General population	STP	54	-	-	[15]
**SA**	Angola	Mbundu	Ang	466	Bantu	-	[17]
		West-Savanna			Bantu	-	[18]
		Cabinda (Fiote)	Cab	109	Bantu	-	[19]
		Kuvale	Kuv	55	Bantu	Seminomadic pastoralists	[20]
		Nyaneka	Nyane	59	Bantu	Agriculturalist	[20]
	Mozambique	General population	Moz	187	-	-	[21,22]
	Zambia	Bisa	Bisa	42	Bantu	-	[23]
		Fwe	Fwe	33	Bantu	Agropastoralist	[24]
		Kunda	Kunda	36	Bantu	-	[23]
		Kwamashi	Kwam	35	Bantu	Agriculturalist	[20]
		Kwangwa	Kwang	35	Bantu	-	[20]
		Lozi	Lozi	110	Bantu	Agriculturalist	[20]
		Mbunda	Mbun	64	Bantu	Agriculturalist	[20]
		Nkoya	Nkoy	32	Bantu	Agriculturalist	[20]
		Tonga	Ton	37	Bantu	Agriculturalist	[24]
	Zimbabwe	Shona	Sho	62	Bantu	-	[6]

References

1. Poloni, E. S. *et al.* Genetic evidence for complexity in ethnic differentiation and history in East Africa. *Annals of Human Genetics* **73,** 582–600 (2009).

2. Boattini, A. *et al.* mtDNA variation in East Africa unravels the history of Afro-Asiatic groups. *American Journal of Physical Anthropology* **150,** 375–385 (2013).

3. Soares, P. *et al.* The Expansion of mtDNA Haplogroup L3 within and out of Africa. *Molecular Biology and Evolution* **29,** 915–927 (2012).

4. Abecasis, G. R. *et al.* An integrated map of genetic variation from 1,092 human genomes. *Nature* **491,** 56–65 (2012).

5. Watson, E., Forster, P., Richards, M. & Bandelt, H. Mitochondrial footprints of human expansions in Africa. *The American Journal of Human Genetics* 691–704 (1997).

6. Castrì, L. *et al.* mtDNA variability in two Bantu-speaking populations (Shona and Hutu) from Eastern Africa: implications for peopling and migration patterns in sub-Saharan Africa. *American Journal of Physical Anthropology* **140,** 302–311 (2009).

7. Krings, M. *et al.* mtDNA Analysis of Nile River Valley Populations: A Genetic Corridor or a Barrier to Migration? *The American Journal of Human Genetics* **64,** 1166–1176 (1999).

8. Tishkoff, S. a *et al.* History of click-speaking populations of Africa inferred from mtDNA and Y chromosome genetic variation. *Molecular Biology and Evolution* **24,** 2180–2195 (2007).

9. Barbieri, C. *et al.* Contrasting maternal and paternal histories in the linguistic context of Burkina Faso. *Molecular Biology and Evolution* **29,** 1213–1223 (2012).

10. Brehm, A., Pereira, L., Bandelt, H. J., Prata, M. J. & Amorim, A. Mitochondrial portrait of the Cabo Verde archipelago: the Senegambian outpost of Atlantic slave trade. *Annals of Human Genetics* **66,** 49–60 (2002).

11. Ely, B., Wilson, J. L., Jackson, F. & Jackson, B. A. African-American mitochondrial DNAs often match mtDNAs found in multiple African ethnic groups. *BMC Biology* **4,** 34 (2006).

12. González, A. M. *et al.* Mitochondrial DNA variation in Mauritania and Mali and their genetic relationship to other Western Africa populations. *Annals of Human Genetics* **70,** 631–657 (2006).

13. Rando, J. C. *et al.* Mitochondrial DNA analysis of Northwest African populations reveals genetic exchanges with European, Near-Eastern, and sub-Saharan populations. *Annals of Human Genetics* **62,** 531–550 (1998).

14. Stefflova, K. *et al.* Evaluation of group genetic ancestry of populations from Philadelphia and Dakar in the context of sex-biased admixture in the Americas. *PloS one* **4,** e7842 (2009).

15. Mateu, E. *et al.* A tale of two islands: population history and mitochondrial DNA sequence variation of Bioko and São Tomé, Gulf of Guinea. *Annals of Human Genetics* **61,** 507–518 (1997).

16. Quintana-Murci, L. *et al.* Maternal traces of deep common ancestry and asymmetric gene flow between Pygmy hunter-gatherers and Bantu-speaking farmers. *Proceedings of the National Academy of Sciences of the United States of America* **105,** 1596–1601 (2008).

17. Plaza, S. *et al.* Insights into the western Bantu dispersal: mtDNA lineage analysis in Angola. *Human Genetics* **115,** 439–447 (2004).

18. Coelho, M., Sequeira, F., Luiselli, D., Beleza, S. & Rocha, J. On the edge of Bantu expansions: mtDNA, Y chromosome and lactase persistence genetic variation in southwestern Angola. *BMC Evolutionary Biology* **9,** 80 (2009).

19. Beleza, S., Gusmão, L., Amorim, A., Carracedo, A. & Salas, A. The genetic legacy of western Bantu migrations. *Human Genetics* **117,** 366–375 (2005).

20. Barbieri, C. *et al.* Unraveling the complex maternal history of Southern African Khoisan populations. *American Journal of Physical Anthropology* **153,** 435–448 (2014).

21. Salas, A. *et al.* The making of the African mtDNA landscape. *The American Journal of Human Genetics* **71,** 1082–1111 (2002).

22. Pereira, L. *et al.* Prehistoric and historic traces in the mtDNA of Mozambique: insights into the Bantu expansions and the slave trade. *Annals of Human Genetics* **65,** 439–458 (2001).

23. De Filippo, C., Heyn, P., Barham, L., Stoneking, M. & Pakendorf, B. Genetic perspectives on forager-farmer interaction in the Luangwa valley of Zambia. *American Journal of Physical Anthropology* **141,** 382–394 (2010).

24. Barbieri, C., Butthof, A., Bostoen, K. & Pakendorf, B. Genetic perspectives on the origin of clicks in Bantu languages from southwestern Zambia. *European Journal of Human Genetics* **21,** 430–436 (2013).

Supplementary Table 7. Haplogroup frequencies and sample size (N) by region and by country/group. Codes for regions: CA – Central Africa, EA – Eastern Africa, SA – Southern Africa, WA – Western Africa. Abbreviations: DRC – Democratic Republic of Congo, Eq. Guinea – Equatorial Guinea, STP – São Tomé and Príncipe.

**Region** Country/Group	**N**	**Haplogroup frequency**																
		**L0a**	**L0d**	**L0k**	**L0***	**L1b**	**L1c**	**L2a**	**L2***	**L3b**	**L3d**	**L3e**	**L3f**	**L3***	**L4**	**L5**	**L***	**M'N(R)**
**WA**	**1941**	**0.014**	**0.000**	**0.000**	**0.000**	**0.128**	**0.041**	**0.232**	**0.173**	**0.117**	**0.064**	**0.099**	**0.026**	**0.012**	**0.004**	**0.000**	**0.000**	**0.089**
Burkina Faso	427	0.023	0.000	0.000	0.000	0.126	0.019	0.272	0.155	0.122	0.056	0.077	0.019	0.009	0.012	0.000	0.000	0.110
Cape Verde	292	0.007	0.000	0.000	0.000	0.079	0.068	0.205	0.209	0.106	0.072	0.147	0.007	0.007	0.000	0.000	0.000	0.092
Gambia	113	0.000	0.000	0.000	0.000	0.115	0.018	0.159	0.265	0.115	0.124	0.088	0.000	0.044	0.018	0.000	0.000	0.053
Ghana	239	0.008	0.000	0.000	0.000	0.096	0.075	0.310	0.088	0.063	0.050	0.197	0.054	0.000	0.000	0.000	0.000	0.059
Mali	305	0.010	0.000	0.000	0.000	0.151	0.030	0.207	0.187	0.138	0.059	0.095	0.030	0.000	0.000	0.000	0.000	0.095
Senegal	293	0.007	0.000	0.000	0.000	0.171	0.020	0.218	0.167	0.147	0.038	0.034	0.017	0.034	0.000	0.000	0.000	0.147
Sierra Leone	272	0.033	0.000	0.000	0.000	0.147	0.059	0.202	0.191	0.118	0.092	0.077	0.048	0.011	0.000	0.000	0.000	0.022
**CA**	**4043**	**0.064**	**0.000**	**0.000**	**0.000**	**0.079**	**0.245**	**0.146**	**0.071**	**0.065**	**0.043**	**0.165**	**0.062**	**0.017**	**0.004**	**0.002**	**0.000**	**0.038**
Cameroon	859	0.081	0.000	0.000	0.000	0.045	0.332	0.108	0.035	0.061	0.049	0.162	0.066	0.013	0.003	0.000	0.000	0.044
Chad	138	0.022	0.000	0.000	0.000	0.225	0.014	0.167	0.109	0.203	0.043	0.080	0.036	0.036	0.014	0.007	0.000	0.043
DRC	39	0.256	0.000	0.000	0.000	0.000	0.000	0.641	0.000	0.000	0.000	0.000	0.000	0.000	0.000	0.103	0.000	0.000
Eq. Guinea	45	0.089	0.000	0.000	0.000	0.000	0.267	0.200	0.044	0.000	0.000	0.244	0.000	0.000	0.000	0.000	0.000	0.156
Gabon	946	0.080	0.000	0.000	0.000	0.054	0.424	0.125	0.050	0.020	0.037	0.145	0.050	0.011	0.005	0.000	0.000	0.000
Niger	233	0.021	0.000	0.000	0.000	0.185	0.013	0.116	0.150	0.240	0.069	0.069	0.034	0.017	0.009	0.000	0.000	0.077
Nigeria	1554	0.046	0.000	0.000	0.001	0.086	0.128	0.168	0.093	0.066	0.042	0.205	0.085	0.024	0.003	0.000	0.000	0.053
Pygmy groups	121	0.074	0.000	0.000	0.000	0.017	0.554	0.182	0.008	0.000	0.025	0.107	0.000	0.000	0.008	0.025	0.000	0.000
STP	108	0.074	0.000	0.000	0.000	0.167	0.194	0.111	0.102	0.046	0.056	0.204	0.028	0.019	0.000	0.000	0.000	0.000
**EA**	**1994**	**0.123**	**0.005**	**0.000**	**0.052**	**0.008**	**0.007**	**0.124**	**0.029**	**0.024**	**0.018**	**0.030**	**0.040**	**0.132**	**0.118**	**0.033**	**0.012**	**0.246**
Ethiopia	648	0.085	0.000	0.000	0.025	0.014	0.002	0.110	0.026	0.008	0.022	0.008	0.037	0.130	0.094	0.068	0.028	0.346
Kenya	570	0.170	0.005	0.000	0.091	0.005	0.005	0.098	0.018	0.037	0.011	0.051	0.053	0.168	0.035	0.023	0.000	0.230
Rwanda	42	0.167	0.000	0.000	0.167	0.000	0.024	0.071	0.048	0.167	0.024	0.071	0.000	0.143	0.071	0.048	0.000	0.000

Supplementary Table 7. (continued)

Somalia	178	0.062	0.006	0.000	0.017	0.017	0.011	0.135	0.112	0.011	0.039	0.028	0.034	0.225	0.039	0.006	0.017	0.242
Sudan	178	0.051	0.000	0.000	0.000	0.006	0.011	0.298	0.045	0.017	0.011	0.006	0.096	0.096	0.045	0.028	0.000	0.292
Tanzania	378	0.177	0.016	0.000	0.066	0.000	0.013	0.106	0.003	0.024	0.016	0.042	0.005	0.056	0.360	0.003	0.008	0.106
**SA**	**2746**	**0.117**	**0.207**	**0.013**	**0.002**	**0.024**	**0.115**	**0.139**	**0.039**	**0.022**	**0.062**	**0.144**	**0.041**	**0.005**	**0.005**	**0.006**	**0.000**	**0.059**
Angola	709	0.171	0.062	0.004	0.000	0.041	0.193	0.103	0.071	0.025	0.051	0.172	0.068	0.007	0.000	0.000	0.000	0.032
Botswana	96	0.177	0.000	0.000	0.000	0.021	0.042	0.240	0.021	0.010	0.104	0.208	0.052	0.000	0.052	0.063	0.000	0.010
Cabinda	110	0.127	0.000	0.000	0.000	0.027	0.245	0.045	0.082	0.036	0.055	0.218	0.127	0.009	0.000	0.000	0.000	0.027
Mozambique	338	0.074	0.062	0.000	0.000	0.015	0.062	0.405	0.036	0.036	0.068	0.180	0.030	0.000	0.000	0.006	0.003	0.024
Namibia	262	0.034	0.424	0.111	0.000	0.011	0.031	0.015	0.008	0.000	0.206	0.080	0.073	0.004	0.004	0.000	0.000	0.000
South Africa	631	0.046	0.597	0.000	0.000	0.006	0.010	0.046	0.008	0.010	0.025	0.032	0.005	0.010	0.008	0.003	0.000	0.195
Zambia	541	0.165	0.026	0.006	0.011	0.031	0.198	0.185	0.048	0.030	0.039	0.214	0.024	0.000	0.006	0.013	0.000	0.006
Zimbabwe	59	0.271	0.017	0.017	0.000	0.034	0.085	0.203	0.034	0.068	0.068	0.186	0.000	0.000	0.017	0.000	0.000	0.000
